# A new rat model of treatment-naive quiescent choroidal neovascularization induced by human VEGF165 overexpression

**DOI:** 10.1242/bio.048736

**Published:** 2020-06-11

**Authors:** Shan Liu, Antje K. Biesemeier, Alexander V. Tschulakow, Harsh V. Thakkar, Sylvie Julien-Schraermeyer, Ulrich Schraermeyer

**Affiliations:** 1 Center for Ophthalmology, Division of Experimental Vitreoretinal Surgery, Tübingen 72076, Germany; 2 Natural and Medical Institute at the University of Tübingen, Applied Material Science and Electron Microscopy, Reutlingen 72770, Germany; 3 STZ OcuTox Preclinical Drug Assessment, Hechingen 72379, Germany

**Keywords:** Choroidal neovascularization (CNV), Vascular endothelial growth factor (VEGF), Electron microscopy (EM), Bevacizumab, Angiogenesis, Age-related macular degeneration (AMD)

## Abstract

Vascular endothelial growth factor (VEGF) is a crucial stimulator for choroidal neovascularization (CNV). Our aim was to develop a reproducible and valid treatment-naive quiescent CNV (i.e. without signs of exudation and with normal visual acuity) rat model by subretinal injection of an adeno-associated virus (AAV)-VEGFA165 vector. The CNV development was longitudinally followed up *in vivo* by scanning laser ophthalmoscopy/optical coherence tomography, fluorescein and Indocyanine Green angiographies and *ex vivo* by electron microscopy (EM) and immunohistochemistry. In total, 57 eyes were analysed. *In vivo*, a quiescent CNV was observed in 93% of the eyes 6 weeks post-transduction. In EM, CNV vessels with few fenestrations, multi-layered basement membranes and bifurcation of endothelial cells were observed sharing the human CNV features. Human VEGF overexpression, multi-layered retinal pigment epithelium (RPE) (RPE65) and macrophages/activated microglia (Iba1) were also detected. In addition, 19 CNV eyes were treated for up to 3 weeks with bevacizumab. The retinal and CNV lesion thickness decreased significantly in bevacizumab-treated CNV eyes compared with untreated CNV eyes 1 week after the treatment. In conclusion, our experimental CNV resembles those seen in patients suffering from treatment-naive quiescent CNV in wet age-related macular degeneration (AMD), and responds to short-term treatment with bevacizumab. Our new model can, therefore, be used to test the long-term effect of new drugs targeting CNV under precisely-defined conditions.

## Abstract (precis)

**Summary:** We developed a new treatment-naive quiescent rat choroidal neovascularization model by VEGF overexpression, allowing the long-term investigation of new drugs.

## INTRODUCTION

Choroidal neovascularization (CNV) is the growth of newly-formed blood vessels from the choriocapillaris (CC) through a rupture in the Bruch's membrane (BM) into the subretinal space. These abnormal blood vessels often leak blood or fluid, damaging the central vision. CNV is a symptom of many ocular diseases, such as wet age-related macular degeneration (AMD) and myopic CNV (Bhutto and Lutty, 2012). The investigations of the cellular and molecular mechanism of CNV are still ongoing. However, vascular endothelial growth factor (VEGF) has proven to be a key stimulator for CNV. Overexpression of VEGF is observed in CNV patients and the laser-induced CNV animal models (Hoerster et al., 2012; Mu et al., 2018; Ryan, 1982; Yi et al., 1997), and it can induce CNV in rabbits, rats and nonhuman primates (Baffi et al., 2000; Julien et al., 2008; Lebherz et al., 2005; Spilsbury et al., 2000; Wang et al., 2003).

VEGF belongs to a highly specific vascular endothelial growth factor family that promotes vascular permeability, extracellular matrix denaturation, vascular endothelial cell proliferation and angiogenesis. Other factors like HIF-1, angiopoietin (ANG)-1, ANG-2 and platelet-derived growth factor-B (PDGF-B) are also involved in the process of neovascularization (Castro et al., 2018). HIF-1 can activate the transcription of multiple target genes associated with angiogenesis, including VEGF, ANG-2 and PDGF-B (Campochiaro, 2013). ANG-2 can promote endothelial cell proliferation and migration if it collaborates with VEGF (Hackett et al., 2002). ANG-1 is an antagonist of ANG-2, inhibiting vascular permeability (Gale et al., 2002). PDGF-B also plays a role in proliferative retinopathies (Mori et al., 2002).

A CNV vessel is formed based on the proliferation and migration of endothelial cells, which is regulated by proangiogenic factors and changes in the extracellular matrix bed (Neve et al., 2014). The CNV vessels are stabilized by pericyte recruitment that suppresses endothelial cell proliferation (Castro et al., 2018). Macrophages also have an essential role in CNV formation, especially the creation of fibrovascular scars, as they secrete a variety of growth factors (including VEGF) (Grossniklaus et al., 2002; Oh et al., 1999; Tahiri et al., 2016).

At the cellular level, CNV has similarities to a wound-healing process including clotting, inflammation, angiogenesis and fibrosis (Kent and Sheridan, 2003; Schlingemann, 2004). Similar growth factors like VEGF and PDGF are involved in both CNV and skin wound healing (Schlingemann, 2004).

The process of CNV formation in wet AMD might be caused by an age-related thickening of BM, which causes a deregulation of retinal pigment epithelium (RPE) transport, atrophy of CC and neuroretinal hypoxia. In this case, the RPE secretes a high level of VEGF and reduces the expression of angiogenesis inhibitors (Schlingemann, 2004). This imbalance of growth factors underlies the CNV formation.

Intravitreal injections of anti-VEGF drugs [e.g. ranibizumab (Lucentis^®^, Genentech/Novartis), bevacizumab (Avastin^®^, Genentech/Roche), aflibercept (Eylea^®^, Regeneron Pharmaceuticals/Bayer)] are used in the clinic, showing benefits including visual acuity improvement and CNV regression in acute wet AMD and other CNV (e.g. myopic CNV) pathologies (Gao et al., 2018; Schmid et al., 2015). Although anti-VEGF therapy is the conventional treatment for CNV, there are still several adverse effects of anti-VEGF drugs, such as bleeding, increased blood pressure, cataract and photoreceptor loss. A report showed that anti-VEGF therapy could lead to geographic atrophy (GA) in about 40% of patients, which was the end stage of wet AMD with no available treatment (Cho et al., 2015). Gemenetzi et al. supported this hypothesis based on the review of the literature on histopathologic animal studies and clinical trials associated with GA and anti-VEGF treatment (Gemenetzi et al., 2017). Kaynak et al. mentioned that this treatment was still indispensable compared with other treatment options, although it increased GA (Kaynak et al., 2018). Another recent study suggested that the mortality of AMD patients who were also diagnosed with acute myocardial infarction increased after anti-VEGF therapy (Hanhart et al., 2018). In addition, bevacizumab can induce retinal vein thrombosis and thrombotic microangiopathy in monkey eyes (Schraermeyer and Julien, 2013).

In 2013, a new type of CNV, treatment-naive quiescent CNV, was discovered by Querques et al. (2013). This is a kind of CNV without signs of exudation which underwent fluorescein angiography (FA), Indocyanine Green angiography (ICG) and optical coherence tomography (OCT) examinations for at least 6 months (Querques et al., 2013). Recently, optical coherence tomography angiography (OCT-A) has proven to be an effective method to diagnose quiescent CNV (Carnevali et al., 2016; Roisman et al., 2016; Treister et al., 2018). By using this technique, quiescent CNV was not only found in early and intermediate AMD, but also in GA (Capuano et al., 2017). However, the mechanism of quiescent CNV conversion to exudation AMD is still unclear. Besides, there is no definite conclusion on whether quiescent CNV should be treated or not (Serra et al., 2019).

Therefore, preclinical research to better understand the biology of AMD and to improve current therapies has to be continued, and new treatment options have to be found.

For these studies, suitable animal models are essential. The laser-induced CNV rodent model is the most common CNV animal model. It is easy to operate and is capable of inducing several CNV lesions per eye, based on the number of laser burns applied. After BM is perforated by laser, inflammation leads to VEGF overexpression and infiltration of blood vessels from the choroid, forming the CNV and recapitulating the main features of wet AMD. However, the healthy retina is damaged due to the burn of the laser before CNV formation starts, and neovascularization may also originate from the retinal vessels (Semkova et al., 2003). In addition, the CNV lesion induced by the laser tends to self-heal within 4 weeks (Giani et al., 2011) so the model can only be used in that time frame. The development of new CNV animal models is therefore ongoing. This study aimed to develop a valid rat CNV model by subretinal injection of the adeno-associated virus (AAV)-VEGFA165 vector to investigate the mechanism of quiescent CNV and new treatment options. We tested whether bevacizumab could stop the conversion of quiescent CNV to exudation AMD, and whether our model would be valid for drug testing. In addition, this model was compared with human CNV samples.

## RESULTS

In this study, the AAV control eyes [AAV-empty vector and AAV-enhanced green fluorescent protein (EGFP) vector] (Table S2 and Fig. S3) did not show any signs of toxicity at any time point after subretinal injections. No CNV-like lesions were found in FA, ICG, OCT and microscopic analyses [light/electron microscopy (LM/EM)] in any eyes after subretinal injection of the AAV-empty vector. The EGFP-vector-transduced eyes showed GFP protein expression in a time frame of 2–9 weeks after subretinal injection. With one exception, the eyes transduced with EGFP vectors did not show any CNV-like lesions in angiographic and histological analyses (data not shown).

### Characterization of the CNV induced by overexpression of VEGF in rat eyes

**Fig. 1.**
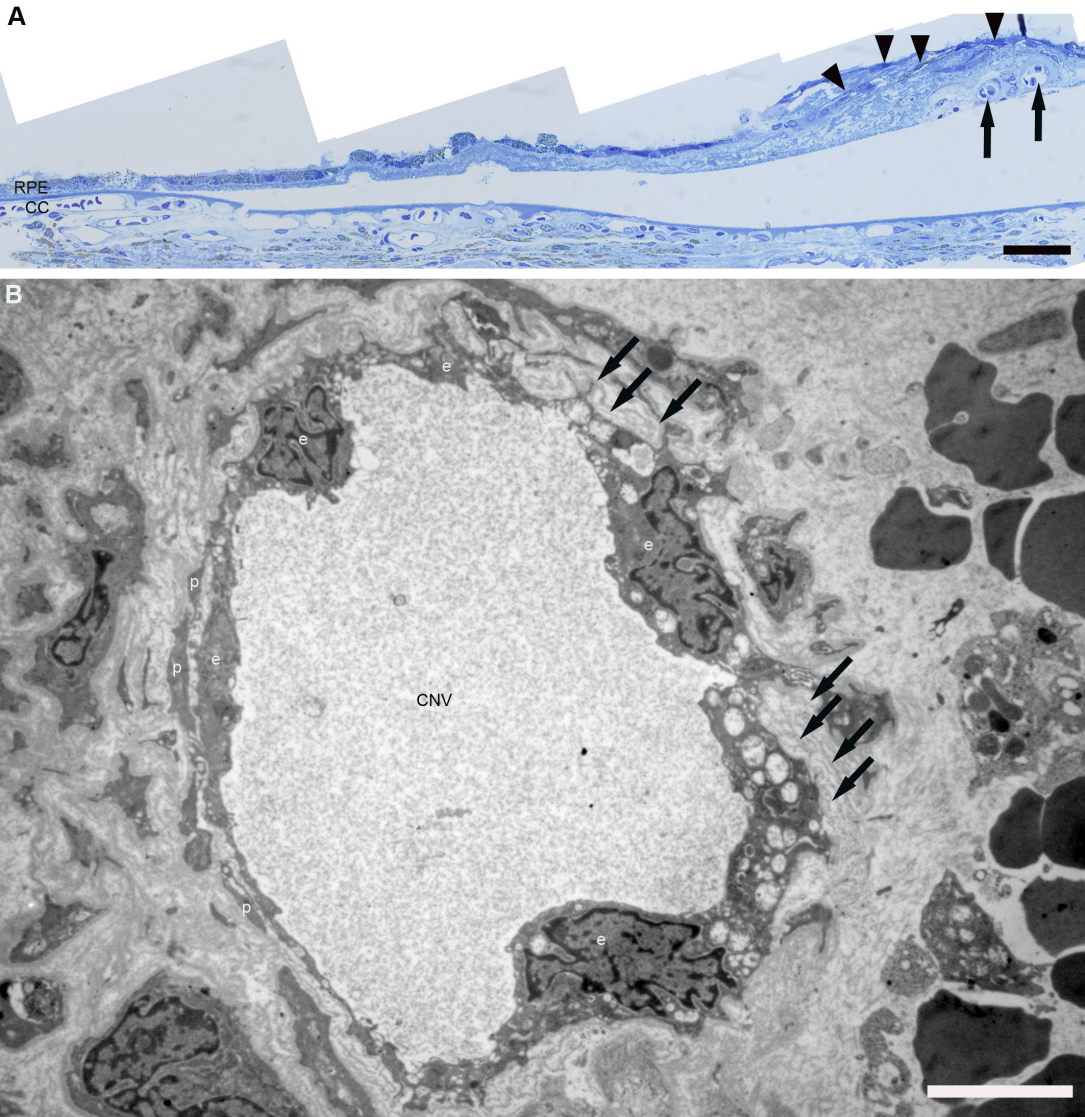
**LM/EM of human CNV.** (A) LM of a human eye with CNV. The multi-layered RPE (arrowheads) is on the right. The space between the multi-layered RPE and CC is filled with the increased extracellular matrix. Black arrows: CNV vessels. (B) EM of a human CNV vessel: varying thicknesses of endothelial cells (e), pericytes (p), multi-layered basement membranes (arrows) and extravascular erythrocytes (right side of B) can be observed. No fenestration was observed. Mitochondria are blown because of preparation artefacts, maybe due to post mortem time. CC, choriocapillaris; CNV, choroidal neovascularization; RPE, retinal pigment epithelium. Scale bar in A: 50 µm, in B: 5 µm.

Human CNV shows irregular hyper-fluorescence in FA/ICG. Subretinal or intraretinal fluid and fibrovascular pigment epithelium detachment are the features of CNV in OCT (Eandi et al., 2017). In addition, the histological changes of human CNV are subretinal neovascularization, migration and proliferation of RPE cells, irregular endothelia of CNV vessels, multi-layered basement membranes, the rupture of BM, leakages, loss of photoreceptors, VEGF overexpression, macrophages/activated microglia deposition, subretinal bleeding and the remodelling of the extracellular matrix (mainly collagen) in BM and the spaces between CNV vessels and RPE cells. Exemplary images of human CNV are presented in Fig. 1.

The eyes successfully transduced with VEGF vector and showing CNV-like signs in FA/ICG/OCT will be termed ‘CNV eyes’ in this paper, and the CNV-like lesion in OCT and the hyper-fluorescent area shown in FA/ICG will be referred to as ‘CNV lesion’ and ‘CNV area’, respectively.

### Angiography and OCT of eyes with CNV

In total, 57 eyes were used to induce CNV in this study. About 50% of the eyes showed hyper-fluorescence in FA/ICG 4 weeks after VEGF vector injection, and 93% of the eyes (including the eyes treated with bevacizumab) showed a CNV in FA 6–9 weeks after VEGF vector transduction (for details see Table S1).

**Fig. 2.**
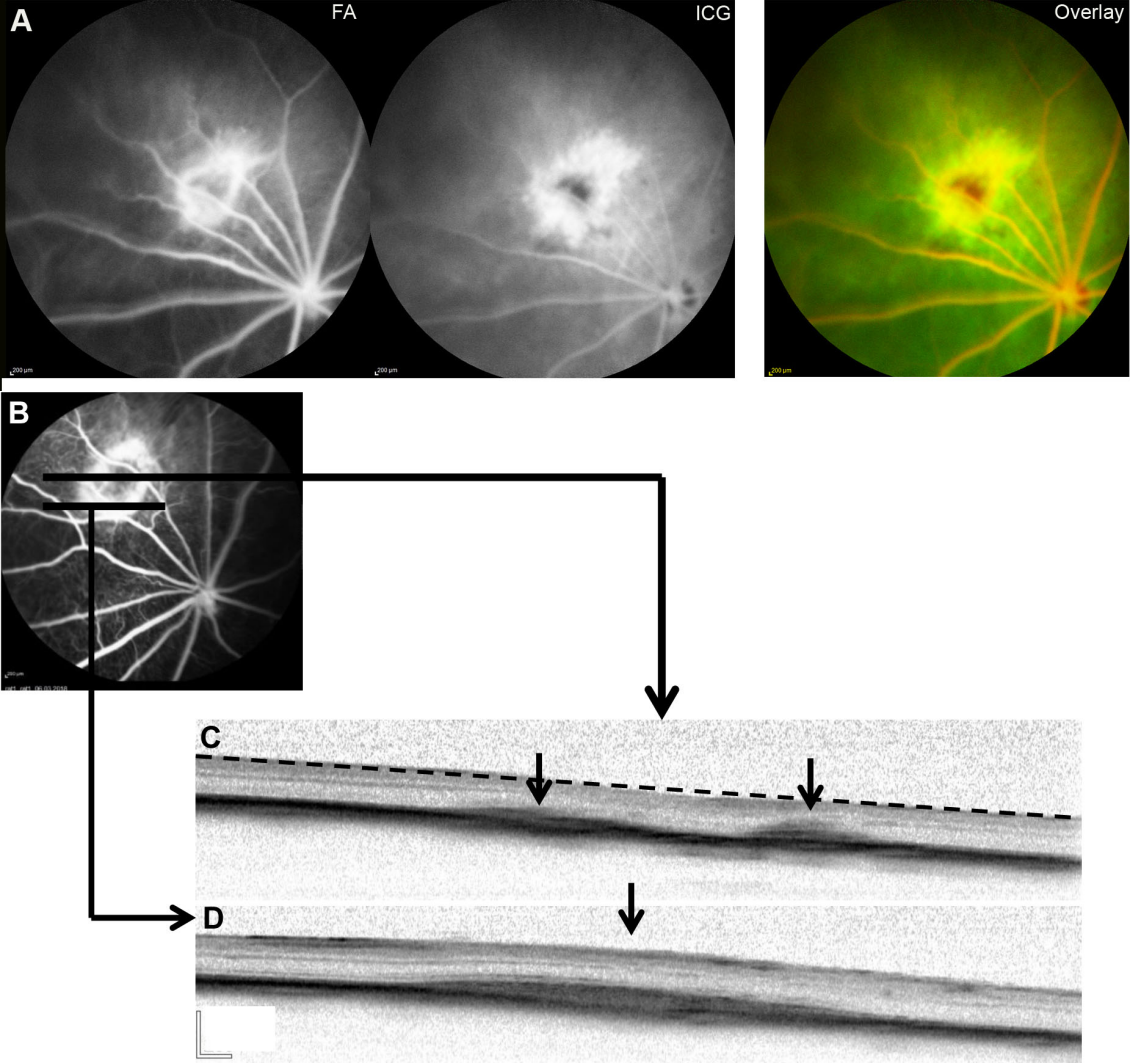
***In vivo* examination of the rat eyes** **6** **weeks after transduction with AAV-VEGF.** (A) Comparison of the CNV area in FA and ICG of an eye. FA and ICG images are on the left, and the overlay image (FA signal: red; ICG signal: green; overlap: yellow) is on the right. The overlapped areas contain the retinal vessels and the CNV area in the retina. The ICG signal shows a spotty pattern stretched over a larger area than the FA signal, indicating the part of CNV below the RPE cells. (B–D) An FA angiograph (B) and corresponding OCT images (C,D) of one eye. The dashed line in C shows the decrease of the retinal thickness between the two small CNV lesions. A large CNV lesion is shown in D, corresponding to the outer rim of the ring-shaped hyper-fluorescent area in FA. No exudation in the subretinal space was found in C and D. Black arrow, CNV lesion. Scale bars: 200 µm.

Because newly-formed CNV vessels extend from the CC through a rupture of the BM into the retina, the vascular changes in the choroid/RPE should only be visible in ICG, and the changes in the retina lead to hyper-fluorescence in both FA and ICG. As Fig. 2 and Fig. S1 illustrate, the CNV eyes always showed a larger hyper-fluorescent area or more lesions in ICG than FA, probably due to CC alterations and occult CNV. A CNV without leakage was shown in all the eyes, since no gradual marked increase in hyper-fluorescence appeared in FA/ICG.

The ring-shaped hyper-fluorescent area with a dark central region is observed in most FA and ICG images (Fig. 2; Fig. S1). The ring-like hyper-fluorescence correlates well with the hyper-reflective areas seen in the OCT images (Fig. 2C,D). Two small CNV lesions in Fig. 2C correspond to the central hyper-fluorescent area in FA (Fig. 2B). No exudation findings in the subretinal space were shown in the OCT images (Fig. 2C,D). The retinal thickness decreased in the dark area compared with the surrounding hyper-fluorescent area in FA.

### LM and EM evaluations of CNV

We mainly focused our EM-analysis on the choroid/RPE interface. The typical features of the RPE cell are the presence of a cell nucleus, pigment granules and microvilli on the apical side. On the basal side, RPE cells have a basal membrane. The RPE layer is normally monolayered and does not have blood vessels. Between the basement membrane of an RPE cell and the basement membrane of CC is a clearly-structured area of extracellular matrix, BM. In the eye, only CC vessels are fenestrated.

**Fig. 3.**
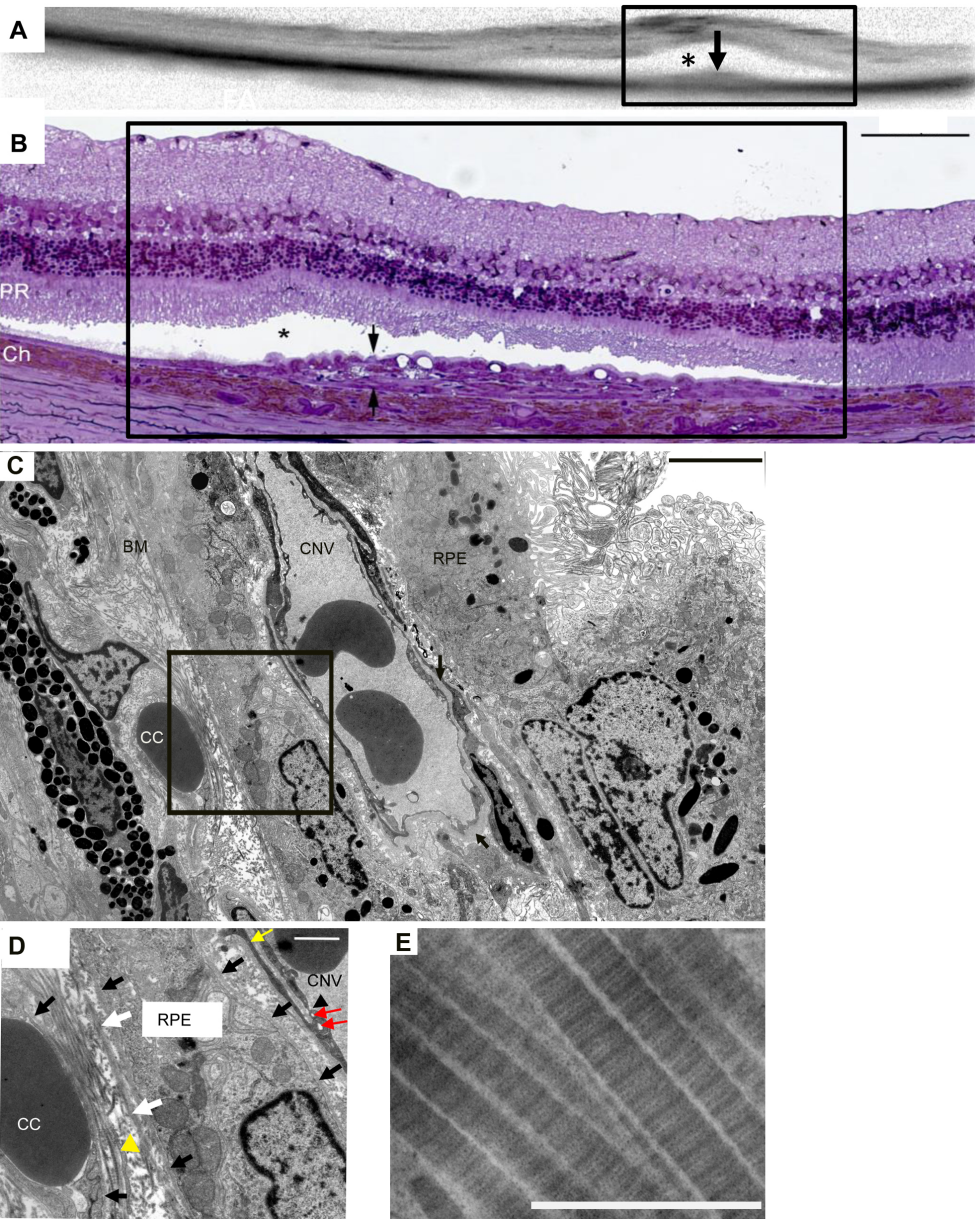
**OCT (A) and LM/EM (B–E) of the same eye, 9** **weeks after VEGF transduction.** (A) The hill-like structure in A (black arrow) corresponds to the CNV area in B and C. *, subretinal space. (B) LM: the black arrows label the CNV area below the subretinal space (*). Large vacuoles and mild photoreceptor degeneration can be observed. The rectangles in A and B show the same region. (C) EM of the same CNV area, a CNV vessel is embedded in the multi-layered RPE. (D) Magnification of the rectangle area in C. Accumulation of collagen with visible striations in BM and between the CNV vessel and the RPE. The elastic layer of BM (white arrow) is incomplete, and a thin cell (yellow arrowhead) can be seen between CC and BM. The bifurcation of endothelial cells (yellow arrow) and abnormal basement membranes surrounding the RPE cells completely can also be observed. Black arrows, basement membranes surrounding CC and RPE; black arrowhead, fenestration; red arrow, pinocytotic vesicles. (E) Collagens are striated with a distinct periodicity. Scale bar in B: 100 µm, in C: 4 µm, in D: 1 µm, in E: 500 nm.

**Fig. 4.**
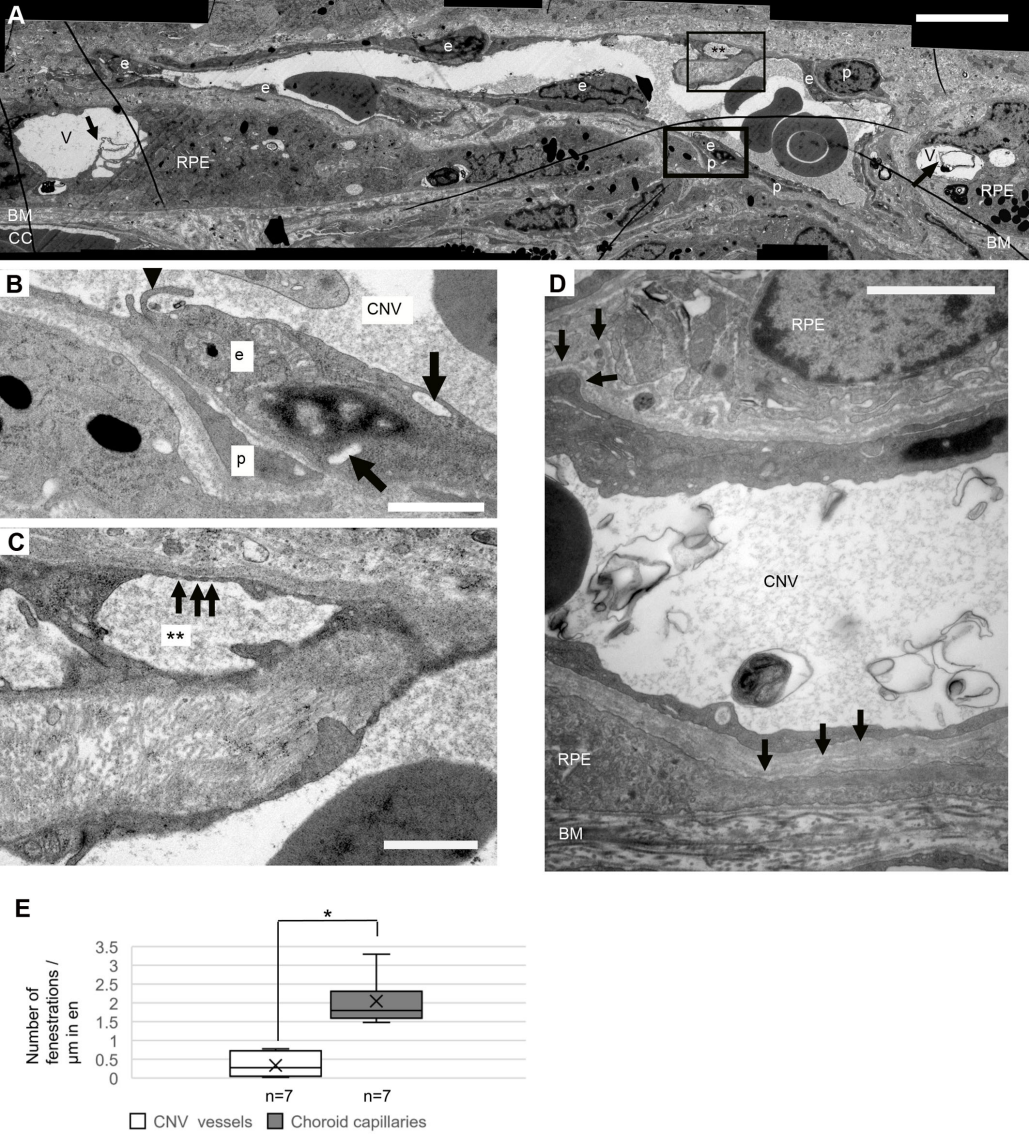
**Ultrastructural details of typical CNV vessels.** (A) A CNV vessel has penetrated through BM (magnified views in B and C). The vessel consists of endothelial cells (e) with varying thicknesses and is associated with pericytes (p). The space between the RPE cells and the CNV vessel is filled with extracellular matrix (mainly collagen) and debris of unknown origin. A small vascular lumen (**) is formed by the irregular endothelium and ‘intrusional’ growth of extracellular matrix (mainly collagen) towards the main lumen of the vessel. Large vacuolar structures (V) in the RPE layer can also be found, they show microvillar projections into the external space (black arrows). A thrombocyte can be observed in the CNV vessel. (B) Magnification of the bottom rectangle area in A. An endothelial cell with several vesicles (marked with black arrows) and a pericyte surrounded by basal membrane can be observed. Black arrowhead, endothelial projections into vessel lumen. (C) Magnification of the top rectangle area in A. Black arrows, fenestrations. (D) Another CNV vessel enveloped by RPE cells. Multi-layered basement membranes (black arrows) can be seen around the vessel. (E) Quantification of the number of fenestrations per µm circumference of the endothelium of CNV vessels and choroid capillaries. The number of fenestrations in the CNV vessels is less than in choroid capillaries (*t*-test, *P<0.05). The mean value and standard deviation are shown in the box figures. Scale bar in A: 5 µm, in B and C: 1 µm, in D: 2 µm.

As mentioned above, FA and ICG angiographs correlate well with the OCT images (Fig. 2). The CNV lesions observed in the OCT images also correlate well with the CNV areas shown in the LM and EM (Fig. 3). As shown in Figs 3 and 4, the CNV induced by VEGF overexpression in rats was characterized by the newly-formed blood vessels with few fenestrations between BM and a multi-layered RPE, loss of photoreceptors and extracellular matrix deposit (mainly collagen, as identified by its distinct ultrastructural striated pattern, Fig. 3E) in BM and the spaces between CNV vessels and RPE cells.

Indeed, not all the CNV vessels showed fenestrations, and they were also fewer fenestrations per vessel as compared to the CC (Fig. 4E, *t*-test, *P*<0.05). The mean number of fenestrations per µm circumference of the endothelium of CNV vessels and choroid capillaries were 0.3±0.3 and 2.0±0.6, respectively. No significant correlation between the size of vessels (neither the CNV vessels nor the CC) and the number of fenestrations was found (data not shown). Fibrin deposition is a sign of the leaky vessels; however, its typical electron-dense shape (Julien et al., 2014) was not found in the CNV eyes.

In addition to the collagen, the proliferation of pigmented RPE-like cells leads to the thickening of the RPE layer towards the retina (Fig. 3B,C). As shown in Fig. 3D, abnormal basement membranes were observed around the RPE cells and the CNV vessels, as well as extracellular matrix deposits in the spaces between RPE and CNV. Indeed, RPE cells usually show basement membranes only on their basal side, but here the basal membranes often surround the whole cellular circumferences. CNV vessels showed both thickened basal membranes (Fig. 3C) and multiple layers of basal membranes (Fig. 4D). Large vacuolar structures formed by the extreme elongation of microvilli were often shown in the RPE layer surrounding the CNV lesion (Figs 3B and 4A).

The restructuring of the extracellular matrix in BM leads to the disruption of RPE cellular function (Fernandez-Godino et al., 2018). Thin cells (elongated undifferentiated cells) were observed in the CNV areas and between CC and BM, which might be an early feature of endothelial proliferation (Fig. 3D). The loss of the elastic layer in BM was often seen in the CNV areas (Fig. 3D). Collagen bundles of different thicknesses appeared disorganized between the CC and BM (Fig. 3D) and in the CNV area. The loss of CC in the CNV eyes was observed in this model (not shown).

A case of invasion of the CNV vessels from the CC to the RPE layer is shown in Fig. 4A. The CNV vessel with a varying thickness of endothelial cells was associated with pericytes (Fig. 4B). The endothelium of the CNV vessels often contained several pinocytotic vesicles (Figs 3D and 4B). The CNV vessel originated from the CC, as it contained fenestrations, which are a typical feature of choroidal capillaries (Figs 3D and 4C). As shown in Fig. 4C, a small vascular lumen was formed by the bifurcations of the endothelium in the CNV vessel. Bifurcation (Fig. 4C) started as endothelial projections pointing into the vessel lumen (Figs 3D and 4B). It is a source of leakage in the neovascular choroidal vessels if the outer endothelial wall closes incompletely (Schraermeyer et al., 2015). Multi-layered basement membranes were often observed around the CNV vessels (Fig. 4D), which is also a feature of human CNV (Schraermeyer et al., 2015).

### Immunohistochemistry (IHC) of CNV eyes

**Fig. 5.**
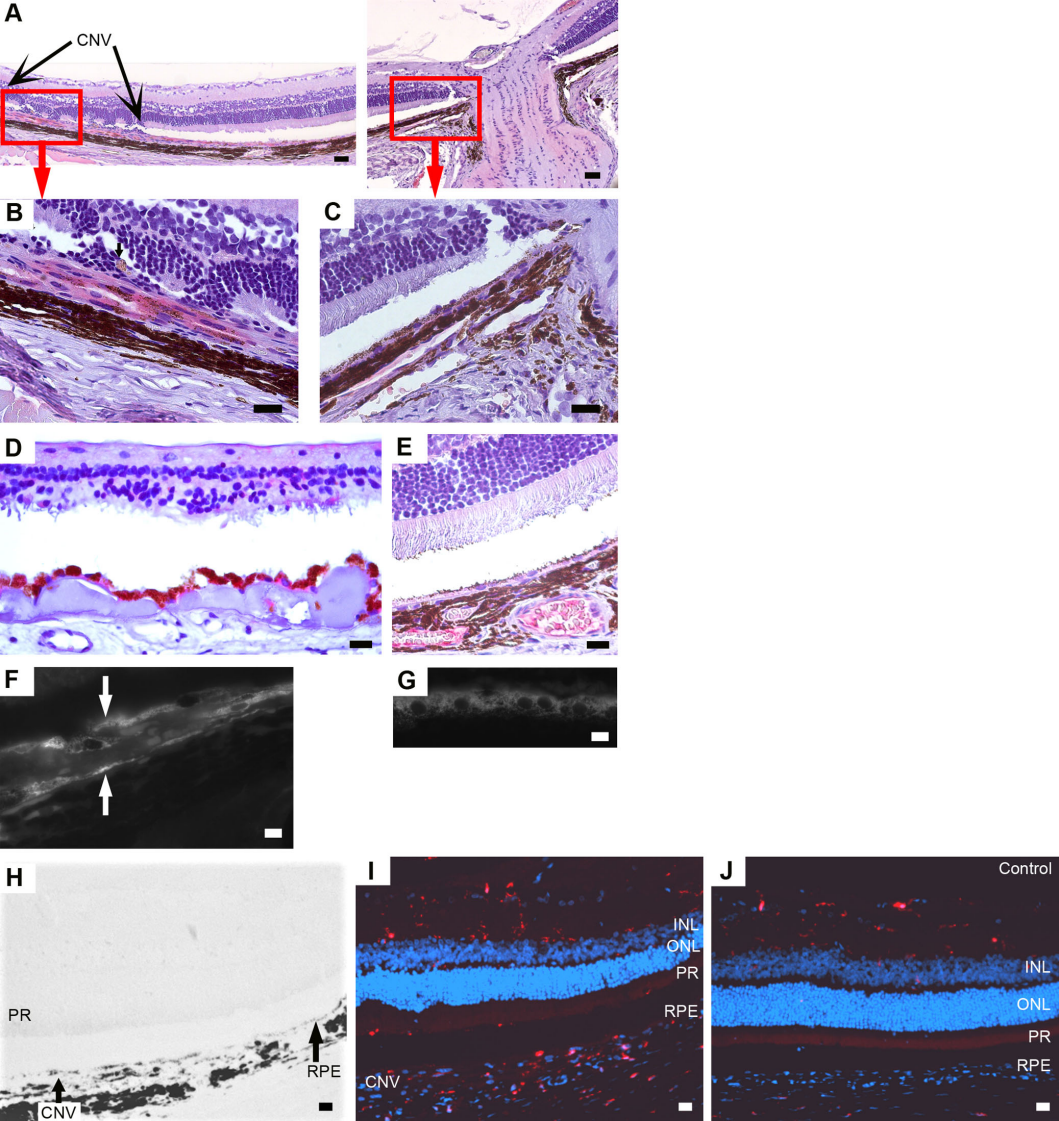
**Exemplary images of anti-human VEGF staining (A–E), RPE65 staining (F–G) and Iba1 staining (with DAPI) (H–J).** (A–E) A CNV rat eye (A–C), a human AMD eye (D) and an AAV-EGFP rat eye (E) with anti-human VEGF staining. (A–C) The CNV rat eye shows an intense anti-human VEGF staining in the RPE cells within and close to the CNV lesions (A,B). The RPE far away from the CNV lesion is not stained significantly (C). A single pigmented cell without VEGF positive stain (marked with a black arrow) can be observed in the subretinal space, which might be a disconnected RPE cell or a macrophage (A). (D) The RPE cells are significantly stained with the human VEGF antibody in the human AMD eye. (E) The AAV-EGFP eye does not show intense VEGF staining in the single RPE layer. (F–G) RPE65: the RPE layer (white arrows) is multiplied in the CNV eye compared with the control in G. The multi-layered RPE corresponds to the pattern of RPE-like pigmented cells under EM. A single layer RPE can be observed in AAV-EGFP control eyes. (H–J) Iba1. (H) Bright field of a CNV rat eye. (I) The same area as in H. The CNV eye shows macrophages/activated microglia deposits in the CNV area and heavily infiltrating the retina. (J) In the AAV-EGFP control eye, no macrophages nor activated microglia in the RPE layer, PR and ONL were observed. INL, inner nuclear layer; ONL, outer nuclear layer; PR, photoreceptors; RPE, retinal pigment epithelium. Scale bar in A: 50 µm, in B–E and H–J: 20 µm, in F and G: 10 µm.

Although VEGF expression is ubiquitous in the retina and the choroid, it is significantly increased in the CNV area, especially in the RPE cells within and close to the CNV lesions (Fig. 5A,B as compared to C). The human AMD eye showed intense VEGF staining in the RPE layer (Fig. 5D), while the RPE cells are not significantly stained with the human VEGF antibody in the AAV-EGFP control eye (Fig. 5E).

The CNV eyes showed multi-layered RPE65 positive staining, indicating that the multi-layered pigmented cells in the CNV lesion were indeed RPE cells (Fig. 5F).

As shown in Fig. 5I, macrophages/activated microglia deposited in the CNV area and heavily infiltrated the retina in the CNV rat eyes with anti-Iba1 staining. No macrophages or activated microglia were observed in the choroid and neural retina in the AAV-EGFP eyes (Fig. 5J).

### Quantification of the CNV areas in angiography and the maximal thickness of the CNV lesion and retina in OCT

**Fig. 6.**
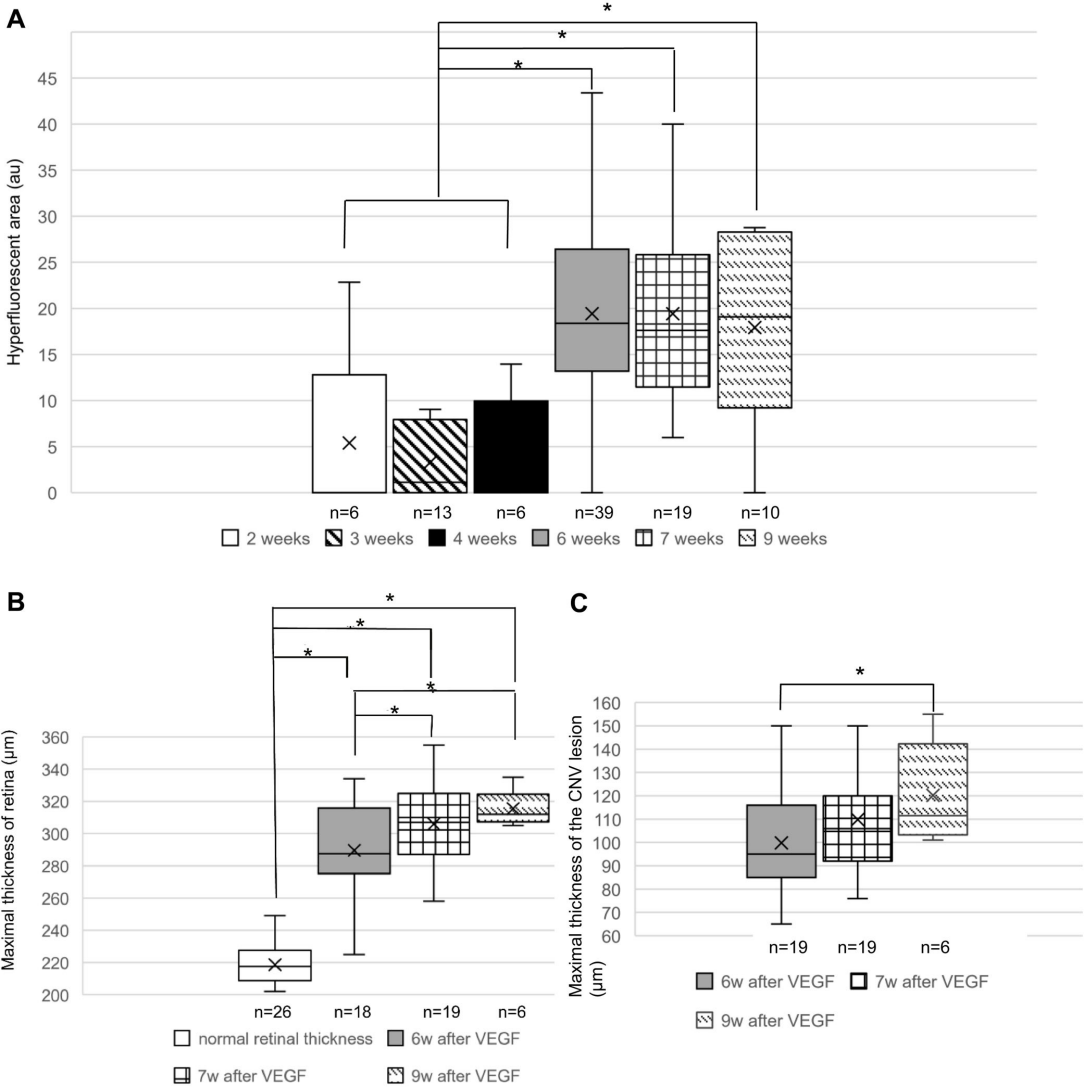
**Quantification of the CNV areas in angiography and the maximal thickness of the CNV lesion and retina in OCT.** (A) Comparison of CNV areas in late-phase FA from 2–9 weeks after VEGF overexpression. The CNV areas reach a stable size after 6 weeks. (B) Quantification of the maximal retinal thickness in CNV eyes from 6–9 weeks after VEGF overexpression. The normal retinal thickness is measured in the adjacent area without CNV lesions. The retina at the CNV area is thicker than the normal retina, and it continues to thicken over time (ANOVA, *P<0.05). (C) Quantification of the maximal CNV lesion thickness in CNV eyes. The CNV lesion thickness increases significantly between 6 and 9 weeks after VEGF transduction (ANOVA, *P<0.05). au=arbitrary units. *P<0.05 (ANOVA). The mean value and standard deviation are shown in the box figures.

As shown in Fig. 6A, the CNV areas in FA angiographs increased significantly after 6 weeks compared with the earlier time points (ANOVA, *P*<0.05; 2 weeks: 5.4±9.3 au, 3 weeks: 3.3±3.7 au, 4 weeks: 4.7±5.7 au, 6 weeks: 19.4±9.8 au, 7 weeks: 19.4±9.3 au, 9 weeks: 17.9±10.0 au). However, there was no significant growth or regression of CNV areas between 6 and 9 weeks after VEGF transduction.

The maximum retinal thickness at the CNV area was much thicker than the normal retinal thickness, and it increased significantly with time (Fig. 6B, ANOVA: *P*<0.05; normal: 218.6±11.4 µm, 6 weeks: 289.6±26.0 µm, 7 weeks: 305.9±24.0 µm, 9 weeks: 315.5±11.0 µm). In particular, the CNV lesion was thickening with time and showed a significant difference if compared between 6 and 9 weeks after VEGF transduction (Fig. 6C, ANOVA: *P*<0.05; 6 weeks: 99.8±19.8 µm, 7 weeks: 109.9±19.6 µm, 9 weeks: 120.2±21.9 µm).

### Treatment effect of bevacizumab in the CNV rat model

FA/ICG did not show statistically significant differences in the area of CNV lesions 6 and 9 weeks after VEGF transduction, either after bevacizumab treatment or without treatment (not shown).

**Fig. 7.**
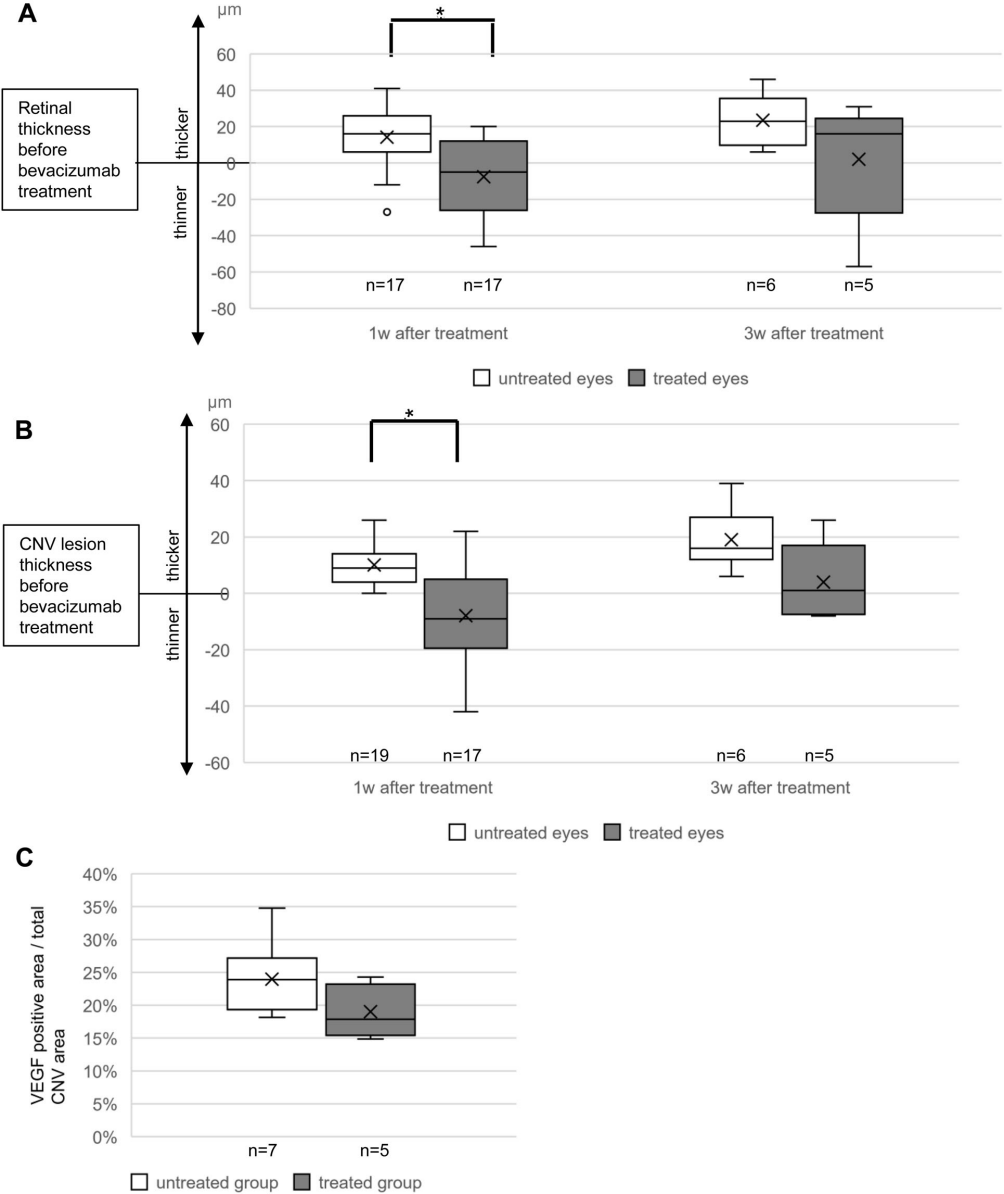
**Change of the maximal retinal (A), CNV lesion thickness (B) and VEGF expression (C) after bevacizumab treatment.** (A,B) Bevacizumab can reduce the retinal and CNV lesion thickness significantly 1 week after treatment (*t*-test, *P<0.05), but the decrease was no longer significant 2 weeks later. (C) The treated eyes did not show a significant decrease in VEGF expression up to 3 weeks after the treatment. CNV, choroidal neovascularization.

The thickness of the retina and CNV lesions decreased significantly 1 week after bevacizumab treatment compared with untreated CNV eyes [Fig. 7A,B, *t*-test, *P*<0.05; treated eyes (retinal thickness changes: −7.6±20.8 µm, CNV lesions changes: −7.9±16.6 µm) versus untreated eyes (retina: 14.2±17.2 µm, CNV lesions: 10.1±7.0 µm)]. The decrease was no longer significant 2 weeks later, but bevacizumab still tended to reduce the growth of CNV.

As the CNV in this rat model was induced by overexpression of human VEGF, the VEGF expression of the bevacizumab-treated eyes and the untreated eyes were compared. The VEGF expression in the treated group showed a slight but not significant decrease compared with the untreated group (Fig. 7C, the percentage of VEGF-positive staining area in the total CNV area: treated group: 19%, untreated group: 24%).

## DISCUSSION

### Correlation of our CNV model to the human CNV with wet AMD

This CNV rat model was developed by overexpression of human VEGFA165, the critical factor for human CNV. A total of 93% of the 57 eyes showed CNV in angiography 6–9 weeks after VEGF vector transduction. The other eyes did not show a CNV, probably due to incorrect operation procedure or the lesion forming outside of the area observable by angiography. In addition, the histologic changes observed in this model greatly mimic early and intermediate human CNV (shown in Fig. 1).

No signs of leakage were presented in FA/ICG/OCT in our model. Overexpression of VEGF leads to the hyper-permeability of the vessels to fibrinogen and the other plasma proteins (Dvorak et al., 1995). Extravasated fibrinogen is rapidly converted into fibrin during the clotting process (Brown et al., 1989; Weisel and Litvinov, 2017). Therefore, fibrin deposition is a sign of a leaky vessel. Fibrin deposition was also not found in EM in our model, implying that the CNV induced in our model is quiescent CNV. Few fenestrations in the endothelium were observed in the CNV vessels of this CNV rat model (Fig. 4E), which correlates with the low number of endothelial fenestrations in human CNV (Biesemeier et al., 2014; Schraermeyer et al., 2015). Hofman's group indicated that the vessel leakage was not caused by the fenestration formation, but was due to the increased active pinocytotic vesicles in the endothelia of CNV vessels (Hofman et al., 2000). There were always several active pinocytotic vesicles in the endothelia in our rat CNV model (Figs 3D and 4B). However, no exudation was seen in our model. The level of VEGF is probably not high enough to stimulate a high number of active pinocytotic vesicles in the endothelia, thus no leakage is observed in our model. If this is the case, inhibiting the expression of VEGF as stabilisation of the newly-formed vessels by pigment epithelium-derived factor (Julien-Schraermeyer et al., 2019) could possibly delay or avoid conversion of quiescent CNV to exudation AMD.

The typical features for the late CNV in wet AMD patients, such as irregular or multi-thrombocytes, complete loss of photoreceptors and loss of endothelia and pericytes, were not shown in our rat CNV model. The CNV vessels induced in this model seem to be more intact and functional than human CNV membranes (Biesemeier et al., 2014), which is probably why the photoreceptors in our model survived. Taken together, these findings imply that the CNV induced in our model is similar to so-called quiescent CNV. Early-stage CNV is difficult to investigate in human CNV membranes, since the sub-macular surgery with excision of the CNV has only been carried out at a very late stage in the past. Recently, Ali et al. (2019) developed a hypoxia-treated zebrafish model to study the early pathological vascular remodelling events of CNV. Our rat model is valid to investigate the whole process of CNV formation, as well as the long-term effects of new treatments, since a CNV induced by AAV-VEGF vector could exist up to 20 months after the vector injection (Wang et al., 2003). In our model, the CNV area reached the maximal size 6 weeks after VEGF transduction (Fig. 6A); thus, the bevacizumab treatment was performed at that point.

### Correlation to the common rodent CNV animal models

The comparison of this CNV rat model and other common rodent CNV models is summarized in Table 1. The common CNV rodent models are based on laser burn, transgenic modifications and subretinal injection of viral vectors (Grossniklaus et al., 2010; Liu et al., 2017; Pennesi et al., 2012).

**Table 1.**
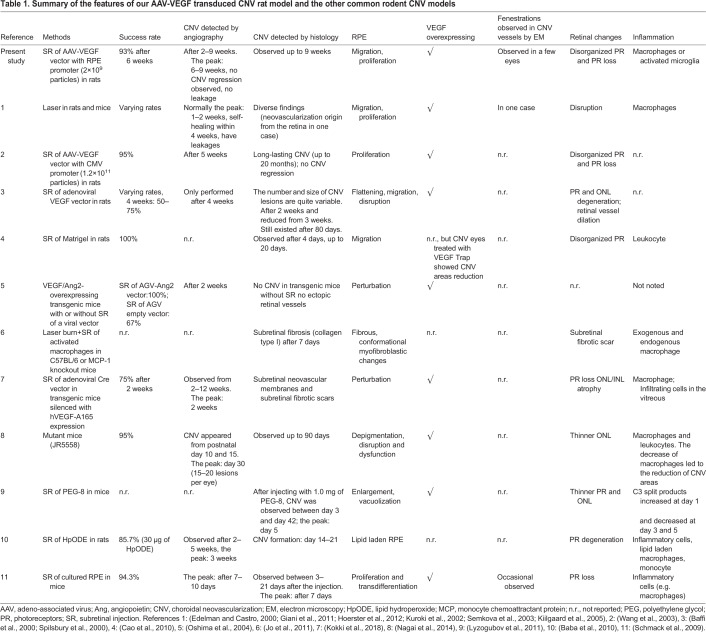
**Summary of the features of our AAV-VEGF transduced CNV rat model and the other common rodent CNV models**

### Correlation to the laser-induced CNV model

The laser-induced CNV animal model (normally rodent) is the most commonly used one (Lambert et al., 2013; Liu et al., 2017). Fully grown CNV lesions with leakages can be obtained 1–2 weeks after the laser injury (Edelman and Castro, 2000). However, the CNV typically heals naturally within 4 weeks after the lasering (Giani et al., 2011). Hoerster et al. indicated that only about 8.5% of lesions had leakage, and the regression of the CNV began after the first week (Hoerster et al., 2012). In 2011, Giani's group demonstrated a CNV formation that reached a peak on day 5 and showed a significant reduction by day 7 (Giani et al., 2011). Additionally, the CNV induced by laser burn, an artificial stimulus, resulted in scar formation within a short time frame of several weeks (Kuroki et al., 2002). Therefore, the laser rodent model is a fast model for late CNV and scar formation, but it is not suitable to investigate quiescent CNV and test the long-term effect of new treatment options.

In addition, the laser models in the earlier studies used several different protocols with different laser wavelengths and applications, showing the varying effects. The neovascularization in the laser models might originate from the retina, not the choroid (Kiilgaard et al., 2005; Semkova et al., 2003). In contrast, our rat model is a true CNV model with a high success rate and less damage to the retina, because the neovessels with fenestrations can be observed in all the CNV eyes.

### Correlation to the transgenic rodent models

The transgenic mice models with overexpression of VEGF and/or ANG-2 in the RPE cells did not display CNV or only induced intrachoroidal neovascularization (Ohno-Matsui et al., 2002; Oshima et al., 2004; Schwesinger et al., 2001). Oshima's group found that rupture to the BM caused by subretinal injection of a vector was important to induce CNV, while an empty vector induced only significantly smaller CNVs compared with ANG-2 vector. They suggested that the subretinal injection of the VEGF vector resulted in the mechanical injury as well as an imbalance of VEGF and its inhibitory factors in the CC-BM-RPE interface (Oshima et al., 2004). In addition, Grossniklaus et al. demonstrated that the trauma caused by the subretinal injection alone might lead to a small-sized CNV (Grossniklaus et al., 2010). Therefore, the break of BM is required to induce subretinal CNV (Schwesinger et al., 2001), and the subretinal injection of viral vectors featuring CNV is a good choice for the development of CNV models.

In this study, a small CNV was found in one EGFP control eye, which is possibly due to the rupture of BM caused by the subretinal injection. Its thickness was not quantifiable due to its small size. Moreover, a scar with small-sized CNV caused by the injection was sometimes found in the CNV eyes in addition to the significant CNV lesion below the subretinal bleb caused by vector volume deposition. This main lesion was investigated in this study to avoid the false interpretation of injection scar tissue.

A report described a transgenic mouse model with a subretinal injection of human VEGF-A165 by adenoviral Cre gene delivery (Kokki et al., 2018). A total of 75% of the mice showed the maximal CNV areas in angiography at 2 weeks after the subretinal injection of VEGF; however, the CNV area began to diminish at later time points. Additionally, retinal atrophy, a feature of late CNV, was observed from 2 weeks after VEGF injection. In our study, CNV induced by an AAV-VEGF vector reaches a peak at 6 weeks after VEGF transduction. Thus, this model provides more sufficient time for the investigation of early CNV than other models and remains valid for investigating CNV over several months.

JR5558 mice, a recently developed spontaneous CNV mouse model, displayed subretinal neovascularization up to postnatal 90 days (Nagai et al., 2014), but later it was proven by other reports that the neovessels originated from the retina (Liu et al., 2017; Hasegawa et al., 2014). To our knowledge, all the animals used for the CNV rodent models are from a very young age, especially the spontaneous CNV mice, which is a limitation of these models. In this AAV-VEGF-induced CNV rat model, we also used young rats; however, much older rats can be investigated, as CNV can exist for more than 20 months (Wang et al., 2003).

### Correlation to the other rodent models

Other CNV rodent models are mainly developed by subretinal injection of Matrigel, an extracellular matrix protein mixture, cells or VEGF vectors. Cao's group developed a rat CNV model by subretinal Matrigel injection in which 100% of the eyes displayed CNV, and VEGF Trap inhibited the growth of CNV (Cao et al., 2010). However, Matrigel created a physical barrier in the subretinal space, while extracellular matrix first deposits in BM in human patients, indicating that the mechanism of this model is not the same as in human patients, and the long-term induction of CNV is not proven.

In another report, macrophage-rich peritoneal exudate cells were subretinally injected into C57BL/6 or MCP-1 knockout mice, to establish a subretinal fibrosis model that resembles advanced AMD (Jo et al., 2011). Note that the mice were treated with a laser to break BM before subretinal injection. The mice showed subretinal fibrosis 7 days after the subretinal injection, indicating that activated macrophages lead to fibrosis. Fibrosis can be defined by the pathological deposit of extracellular matrices (mainly collagen) in the wound healing (Neary et al., 2015). In our study, macrophages and activated microglia deposited in the CNV area (as shown in Fig. 5I), stimulated the formation of fibrosis in this model (see Fig. 3). Subretinally-injected cultured RPE developed CNV in 94.3% of the eyes; however, the CNV regressed from 7 days after the injection (Schmack et al., 2009). This suggested that increased angiogenic factor expression by additional RPE cells could not induce long-term CNV. This could be explained by Stern's group (Stern and Temple, 2015), who demonstrated that RPE proliferation could stimulate CNV regression in the laser-induced CNV model and younger wet-AMD patients through RPE wound repair. The peak of the CNV induced by subretinal injection of polyethylene glycol-8 in mice was 5 days after the injection, and 3 weeks for the CNV induced by lipid hydroperoxide in rats (Baba et al., 2010; Lyzogubov et al., 2011). These models induced by subretinal injection of cells do not seem to be suitable for the long-term investigation of CNV, as CNV regression occurs within 1 and 3 weeks (Table 1).

Several CNV models were induced by viral overexpression of VEGF (Baffi et al., 2000; Spilsbury et al., 2000; Wang et al., 2003). In the last decade, a range of viral vector systems have been used for ocular overexpression of proteins in a lot of studies. Subretinal injection of adenoviral-VEGF vector induced CNV after 4 weeks, and the CNV still existed at 80 days (Baffi et al., 2000; Spilsbury et al., 2000). However, Campochiaro and Zhang et al. mentioned that the adenoviral vector system was active with high immunogenicity for only about 1 month, and the vector itself has high retinal toxicity (Campochiaro, 2011; Zhang et al., 2012). The CNV in the adenovirus VEGF models was partly induced by the inflammatory responses to the adenovirus vector itself (Baffi et al., 2000; Spilsbury et al., 2000). In contrast, the AAV vector did not lead to inflammatory responses (Fisher et al., 1997; Wang et al., 2003). The AAV7 and AAV8 vectors have been tested by Xiong's team, who found that the toxicity of AAV was associated with certain AAV cis-regulatory sequences (Xiong et al., 2019), which suggested that the ocular AAV toxicity might not be due to the AAV vector but the DNA contained in it. The AAV2 was used in our study. It is still a good vector, because the AAV-empty vectors and EGFP vectors did not show any retinal toxicity in our study, and the CNV induced by the AAV-VEGF vector can exist for a very long period (Rolling et al., 2006; Wang et al., 2003). Therefore, our model can be used to investigate the whole process of CNV formation, especially the early CNV that cannot properly be studied in the laser model, as well as the human donor tissues.

In the Wang et al. study, they used the AAV-VEGF vector with CMV promotor and investigated the rat model over a long timeframe of 5 weeks to 20 months after VEGF transduction. CNV was shown in 95% of the eyes; however, only one to three eyes were investigated for each time point.

In our study, an AAV-VEGF vector with a specific RPE promotor was developed, and the earlier time points of this model were investigated (2–9 weeks). The vector used in this study has proven to be efficient, as 93% of the eyes injected with about 60 times less viral particles than were used in Wang's study showed CNV 6 weeks after VEGF transduction. Additionally, the subretinal neovascularization displayed in this model originates from the CC, as the fenestrations observed in the CNV vessels are a feature of choroidal capillaries. As shown in Table 1, only a few reports proved that the subretinal neovascularization found in their models were truly from the CC. To our knowledge, our rat model is the first model that can be used to investigate treatment-naive quiescent CNV.

As there is usually only one lesion per eye in the AAV-VEGF transduced CNV model, it is difficult to quantify the ultrastructural features, which is a limitation of that model. In contrast, the laser model has more lesions to be investigated in each eye (usually three to five individual lesions) and a spontaneous CNV model also has multiple lesions per eye (15–20 lesions per eye).

### Treatment of CNV in the CNV rat model using bevacizumab

Bevacizumab can directly interact with VEGF extracellularly, avoiding the combination of VEGF and VEGFRs, and inhibiting the progression of CNV. It is commonly used in the clinic due to its extremely low price in comparison with the other anti-VEGF drugs. Therefore, bevacizumab was used in this study to verify that this CNV rat model is valid for the evaluation of treatment strategies for quiescent CNV.

As 6 weeks were needed to reach a full-grown CNV in this CNV rat model, bevacizumab was injected into the CNV eyes at that time point. A report indicated that the conversion of quiescent CNV to exudation AMD showed a preferential increase of the thickness of the CNV lesions rather than the diameter of the CNV lesions (Serra et al., 2019). In our study, the thickness of the CNV lesions decreased significantly after a 1-week treatment, thereby inhibiting the conversion of quiescent CNV to exudation AMD. However, bevacizumab did not show an apparent effect after 3 weeks of treatment. This also often happens in human AMD patients; therefore, they need further reinjection of bevacizumab.

The expression of VEGF analysed by human VEGF staining decreased in bevacizumab-treated rat eyes; however, this was not statistically significant compared with untreated eyes. There is no intense human VEGF staining in the RPE layer of the AAV-EGFP eye, or in the RPE distant from the CNV lesion of the CNV eyes (shown in Fig. 5C,E). Therefore, it seems that the anti-human VEGF antibody mainly recognizes human VEGF. The overexpression of human VEGF in our model leads to the thickening of BM and the RPE layer. These changes also result in the atrophy of CC and neuroretinal hypoxia, which promotes the upregulation of rat VEGF secreted by RPE cells. This is also one reason why the area of CNV lesion did not decrease significantly after being treated with bevacizumab.

One reason for the insignificant VEGF staining changes after treatment is that bevacizumab can only inhibit the expression of VEGF in a short time frame, as the plasma-free VEGF level markedly decreased within the first week after bevacizumab injection in human AMD patients, but later it increased again (Avery et al., 2017). Another reason is that VEGF is still overexpressed in the CNV eyes, as the untreated CNV eyes show ongoing CNV formation with a significant increase of retinal and CNV lesion thickness up to 9 weeks after VEGF vector transduction (Fig. 6). Future studies may benefit from using inducible vectors that can be switched on and off according to the experimental needs.

Pachydaki et al. indicated that stable CNV vessels with pericyte support did not respond to bevacizumab, and only degenerating, leaky vessels are targeted by bevacizumab (Pachydaki et al., 2012). This finding can also contribute to the insignificant treatment efficacy of bevacizumab in this CNV rat model with foremost pericyte-containing CNV vessels and absent leaky vessels.

Additionally, it was also demonstrated that bevacizumab did not show a significant therapeutic effect in the laser-induced rodent CNV model (Lu and Adelman, 2009), as bevacizumab was unable to bind to murine VEGF with high affinity (Fuh et al., 2006; Yu et al., 2008). Non-human primate laser-induced CNV models can be used to test antiangiogenic treatments (Husain et al., 2005; Krzystolik et al., 2002; Lichtlen et al., 2010); however, it is much more expensive and difficult to obtain permission to use a large number of non-human primates. Therefore, the rat CNV model induced by overexpression of human VEGF may be an ideal alternative to test the anti-human VEGF therapy.

This model could also be used to study the formation of new healthy choroidal blood vessels in quiescent CNV. Such intact vessel formations have recently been suggested to have a protective effect in inhibiting the growth of GA (Heiferman and Fawzi, 2019). If the remodelling of choroidal vessels were better understood, replacement of degenerating CC regulated by growth factors in human patients might become possible.

In summary, our rat model resembles human quiescent CNV in AMD, based on *in vivo* imaging and histologic examinations, especially at the ultrastructural level. Bevacizumab tends to inhibit the conversion of quiescent CNV to exudative AMD in the short term. Therefore, this CNV model is valid to test new drugs for quiescent CNV. Regarding the disadvantages of the laser-induced model, the CNV model induced by subretinal injection of the AAV-VEGF vector is a good choice, as VEGF is a major cause of human CNV. In addition, it can be used to better understand the underlying mechanisms of CNV formation and help to develop new therapy options.

## MATERIALS AND METHODS

### Animals

7-week-old female Long Evans rats were purchased from Janvier Labs, Le Genest-Saint-Isle, France. There was no significant difference in gender for the morbidity of human CNV (Colijn et al., 2017); therefore, only female rats were used because of their docile behaviour. In total, 66 eyes were used in this study (for details see Table S1).

The animal experiments were performed after approval by the Regierungspräsidium Tübingen (AK 09/14). All of the animals were handled in conformity to the German Animal Welfare Act and were under the control of the animal protection agency and supervision of veterinarians of the University of Tübingen.

### CNV from human eyes

Five human CNV samples removed during sub-macular surgery were analysed by LM/EM and IHC (the same samples used in Biesemeier et al., 2014; Schraermeyer et al., 2015).

The study of human CNV membranes followed the guidelines of the Declaration of Helsinki and was approved by the Ethics Committee of the University of Tübingen. Each patient gave written consent for the scientific use of the specimens. Some of the eyes were a gift from The Foundation Fighting Blindness Eye (FFB) Donor Program (Columbia, MD, USA); others were provided by the University Eye Hospital Tübingen (ethical number for scientific issues 462/2009BO2) (Biesemeier et al., 2014; Schraermeyer et al., 2015).

### AAV-vector system

The vectors were produced by Sirion Biotech GmbH (Munich, Germany). AAV-VEGF-A vector was used to induce CNV in this rat CNV model. As shown in Fig. S2, human VEGF-A165 cDNA, from the plasmid pBLAST49-hVEGF, was inserted in an AAV2 vector (subtype 4) backbone. An RPE specific promotor RPE65 was used. EGFP was added instead of human VEGF-A165 in AAV-EGFP vector. Empty AAV vector without any expression cassette and AAV-EGFP vector served as controls.

### Study design

The experimental design of the eyes injected with AAV-EGFP or AAV-empty vectors is shown in Fig. S3A. One AAV-EGFP eye was enucleated 9 weeks after the vector subretinal injection, and the residual eyes were enucleated 4 weeks after the vector transduction (see Table S2). As shown in Fig. S3B, in order to study the processes of CNV formation, *in vivo* imaging examinations were performed at different time points after VEGF vector injection (2–9 weeks), and the eyes were enucleated at different time points (4, 6, 7 and 9 weeks). The waiting time to reach the maximal CNV area was confirmed by *in vivo* imaging analysis, which was also the best time point for testing the treatments. Therefore, the bevacizumab treatment was performed 6 weeks after the VEGF transduction, and the time flow overview of the experiment was shown in Fig. S3C. All eyes were enucleated for IHC or LM/EM analysis, directly after the last *in vivo* imaging session. The number of eyes with quantifiable data is less than that shown in Table S1, due to the death of rats during the examination or severe cataract and bleeding, which made imaging impossible.

### Subretinal injection

The subretinal injection was performed according to the previous work of Julien et al. (2008). 2 µl of vector suspension (1×10^9^ particles/µl) was injected into the subretinal space of the eye.

### Intravitreal injection of bevacizumab

5 µl solution was delivered through the pars plana into the vitreous cavity by using a NanoFil 34-gauge bevelled needle (Hamilton Co., Reno, NV, USA). Nineteen eyes were intravitreally injected with 5 µl bevacizumab (25 mg/ml, bevacizumab, Roche, Basel, Switzerland) 6 weeks after subretinal injection of AAV-VEGF-A165 vector in this study.

### *In vivo* imaging

Scanning laser ophthalmoscopy (SLO), FA, ICG and OCT were performed using a Spectralis™ HRA+OCT (Heidelberg Engineering, Heidelberg, Germany) device modified for use with rats based on protocols from other studies (Huber et al., 2009; Fischer et al., 2009). FA and ICG dyes [42 µl fluorescein (Alcon 10%), 208 µl 0.9% NaCl, 250 µl Indocyanine Green (5 mg/ml, Diagnostic Green)] were injected in the tail vein, and *in vivo* imaging was performed using 488 and 795 nm lasers, respectively. Late phase angiograms were acquired after about 10–20 min. The GFP expression was visualized by imaging in the FA mode before the injection of the dyes.

### LM/EM

The eyes were fixed in 5% glutaraldehyde in 0.1 M cacodylate buffer (pH 7.4) overnight at 4°C. The samples were post-fixed with 0.1% osmium tetroxide and then dehydrated with a series of ethanol. The block staining with saturated uranyl acetate in 70% ethanol was performed overnight at 4°C. The samples were further dehydrated to 100% dry ethanol and propylene oxide and finally embedded in Epon (SPI-Pon™812 Epoxy Embedding Kit, SPI Supplies, West Chester, PA, USA). The chemicals for the embedding were purchased from Fluka, Germany.

Semi-thin sections (0.7 µm thick) were stained with Toluidine Blue and observed under LM (Axioplan2 imaging^®^, Zeiss, Göttingen, Germany). Ultra-thin sections (0.05 µm) were stained with lead citrate and examined by EM with a Zeiss 900 transmission electron microscopy (Zeiss, Jena, Germany).

### IHC

After enucleation of the eyes, the whole eyes were fixed in 4.5% formalin (Roti Histofix, Carl Roth, Karlsruhe, Germany) and embedded in paraffin for histologic study. Sections 4 µm thick were cut and then stained with Hematoxylin and Eosin (H&E) or respective primary antibodies: 1:300 mouse anti-human VEGF-A antibody (GeneTex/Biozol, Eching, Germany), 1:100 goat anti-RPE65 antibody (sc-33294; Santa Cruz Biotechnology, Santa Cruz, CA, USA), and 1:1000 rabbit anti-Iba1 antibody (FUJIFILM Wako, Dusseldorf, Germany). H&E staining was performed to locate the CNV area. For most experiments, the DAKO REAL™ Detection System, Alkaline Phosphatase/RED, Rabbit/Mouse kit was used as a secondary antibody. A Cy3 mouse secondary antibody was used as the secondary antibody for the anti-RPE65 antibody staining, and a Cy3 goat secondary antibody was used for the anti-Iba1 staining (secondary antibodies were purchased from Jackson ImmunoResearch, Ely, UK). Sections were cover-slipped with FluorSave (Calbiochem, La Jolla, CA, USA). A Zeiss Axioplan2 imaging microscope (Zeiss, Jena, Germany) was used to investigate the fluorescence staining of the samples.

### Detection and quantification of hyper-fluorescent CNV lesion areas in angiography data sets

According to Lu and Adelman's study (Lu and Adelman, 2009), the area of the hyper-fluorescent region in angiograms corresponds to the area of CNV lesion. The area of hyper-fluorescent regions in FA and ICG angiographs can be measured by contouring the CNV lesion using the software included in the Heidelberg Engineering SLO/OCT device. As the Heidelberg Engineering SLO/OCT machine is designed for humans, the dimensions in the x and y-axes are not calibrated for the animal experiments. In contrast, the dimensions in the z-axis in OCT images are displayed properly. Thus, the hyper-fluorescent areas in angiographs were displayed in arbitrary units (au) in this study.

### Quantification of the maximal thickness of the CNV lesion area in OCT data sets

A series of OCT images in the whole CNV lesion area were collected for each eye in this study by using the volume scan function of the OCT machine. The CNV lesion in each OCT image corresponds to the hyper-fluorescent region in the FA angiograph (see Fig. S4).

As shown in Fig. S5, the thickness of the retina (between internal limiting membrane and choroid) and the CNV lesion was measured in each OCT image of each eye. The maximal thickness of the CNV lesion of each eye was obtained for data analysis. In addition, retinal thickness in the adjoining area without lesion in the OCT image with the maximal retinal thickness was measured as controls and termed as ‘normal retinal thickness’.

### Quantification of fenestrations

The number of fenestrations per µm circumference of the endothelium of CNV vessels and choroid capillaries was quantified in EM images with 30,000× magnification, according to the study of Biesemeier et al. (2014). Seven CNV vessels of this rat model and seven choroid capillaries of the AAV-EGFP transduced eyes were used for this quantification. As not all the CNV vessels contained fenestrations, only the CNV vessels with fenestrations were measured.

### Quantification of VEGF expression in the eyes with CNV

The positive anti-human VEGF stained area and the total CNV area in the eyes with CNV were measured by using ImagePro Plus 6.0 software (Media Cybernetics, MD, USA), as shown in Fig. S6. The percentage of VEGF positive staining area of the total CNV area was calculated to quantify the VEGF expression according to other research (Albaayit et al., 2016; Johansson et al., 2001).

### Statistics

All statistical analyses were performed using IBM SPSS Statistics 25 software (IBM, New York, USA; obtained from University of Tübingen). Student's *t*-test (two-tailed) was performed to compare the results of two normally-distributed groups, and one-way ANOVA was used for multiple comparisons of different groups. The significance level is *P*=0.05. The mean value and standard deviation were shown in the box figures.

## Supplementary Material

Supplementary information

## References

[BIO048736C1] Albaayit S. F. A., Abba Y., Abdullah R. and Abdullah N. (2016). Prophylactic effects of Clausena excavata Burum. f. leaf extract in ethanol-induced gastric ulcers. *Drug Des. Dev. Ther.* 10, 1973-1986. 10.2147/DDDT.S103993 PMC4914073 27366052

[BIO048736C2] Ali Z., Mukwaya A., Biesemeier A., Ntzouni M., Ramsköld D., Giatrellis S., Mammadzada P., Cao R., Lennikov A., Marass M. et al. (2019). Intussusceptive vascular remodeling precedes pathological neovascularization. *Arterioscler. Thromb. Vasc. Biol.* 39, 1402-1418. 10.1161/ATVBAHA.118.312190 31242036 PMC6636809

[BIO048736C3] Avery R. L., Castellarin A. A., Steinle N. C., Dhoot D. S., Pieramici D. J., See R., Couvillion S., Nasir M. A., Rabena M. D., Maia M. et al. (2017). Systemic pharmacokinetics and pharmacodynamics of intravitreal aflibercept, bevacizumab, and ranibizumab. *Retina* 37, 1847-1858. 10.1097/IAE.0000000000001493 28106709 PMC5642319

[BIO048736C4] Baba T., Bhutto I. A., Merges C., Grebe R., Emmert D., Mcleod D. S., Armstrong D. and Lutty G. A. (2010). A rat model for choroidal neovascularization using subretinal lipid hydroperoxide injection. *Am. J. Pathol.* 176, 3085-3097. 10.2353/ajpath.2010.090989 20395434 PMC2877867

[BIO048736C5] Baffi J., Byrnes G., Chan C. C. and Csaky K. G. (2000). Choroidal neovascularization in the rat induced by adenovirus mediated expression of vascular endothelial growth factor. *Invest. Ophthalmol. Vis. Sci.* 41, 3582-3589. 11006256

[BIO048736C6] Bhutto I. and Lutty G. (2012). Understanding age-related macular degeneration (AMD): relationships between the photoreceptor/retinal pigment epithelium/Bruch's membrane/choriocapillaris complex. *Mol. Aspects Med.* 33, 295-317. 10.1016/j.mam.2012.04.005 22542780 PMC3392421

[BIO048736C7] Biesemeier A., Taubitz T., Julien S., Yoeruek E. and Schraermeyer U. (2014). Choriocapillaris breakdown precedes retinal degeneration in age-related macular degeneration. *Neurobiol. Aging* 35, 2562-2573. 10.1016/j.neurobiolaging.2014.05.003 24925811

[BIO048736C8] Brown L. F., Dvorak A. M. and Dvorak H. F. (1989). Leaky vessels, fibrin deposition, and fibrosis: a sequence of events common to solid tumors and to many other types of disease. *Am. Rev. Respir. Dis.* 140, 1104-1107. 10.1164/ajrccm/140.4.1104 2478057

[BIO048736C9] Campochiaro P. A. (2011). Gene transfer for neovascular age-related macular degeneration. *Hum. Gene. Ther.* 22, 523-529. 10.1089/hum.2011.050 21443427 PMC3081438

[BIO048736C10] Campochiaro P. A. (2013). Ocular neovascularization. *J. Mol. Med. (Berl.)* 91, 311-321. 10.1007/s00109-013-0993-5 23329331 PMC3584193

[BIO048736C11] Cao J., Zhao L., Li Y., Liu Y., Xiao W., Song Y., Luo L., Huang D., Yancopoulos G. D., Wiegand S. J. et al. (2010). A subretinal matrigel rat choroidal neovascularization (CNV) model and inhibition of CNV and associated inflammation and fibrosis by VEGF trap. *Invest. Ophthalmol. Vis. Sci.* 51, 6009-6017. 10.1167/iovs.09-4956 20538989 PMC3061520

[BIO048736C12] Capuano V., Miere A., Querques L., Sacconi R., Carnevali A., Amoroso F., Bandello F., Souied E. H. and Querques G. (2017). Treatment-naïve quiescent choroidal neovascularization in geographic atrophy secondary to nonexudative age-related macular degeneration. *Am. J. Ophthalmol.* 182, 45-55. 10.1016/j.ajo.2017.07.009 28734811

[BIO048736C13] Carnevali A., Cicinelli M. V., Capuano V., Corvi F., Mazzaferro A., Querques L., Scorcia V., Souied E. H., Bandello F. and Querques G. (2016). Optical coherence tomography angiography: a useful tool for diagnosis of treatment-naïve quiescent choroidal neovascularization. *Am. J. Ophthalmol.* 169, 189-198. 10.1016/j.ajo.2016.06.042 27394033

[BIO048736C14] Castro P. R., Barbosa A. S., Pereira J. M., Ranfley H., Felipetto M., GONçalves C. A. X., Paiva I. R., Berg B. B. and Barcelos L. S. (2018). Cellular and molecular heterogeneity associated with vessel formation processes. *BioMed Res. Int.* 2018, 1-32. 10.1155/2018/6740408 PMC6199857 30406137

[BIO048736C15] Cho H. J., Lee T. G., Han S. Y., Kim H. S., Kim J. H., Han J. I., Lew Y. J. and Kim J. W. (2015). Long-term visual outcome and prognostic factors of Intravitreal anti-vascular endothelial growth factor treatment for retinal angiomatous proliferation. *Graefes Arch. Clin. Exp. Ophthalmol.* 254, 23-30. 10.1007/s00417-015-2993-3 25825231

[BIO048736C16] Colijn J. M., Buitendijk G. H. S., Prokofyeva E., Alves D., Cachulo M. L., Khawaja A. P., Cougnard-Gregoire A., Merle B. M. J., Korb C., Erke M. G. et al. (2017). Prevalence of age-related macular degeneration in Europe: the past and the future. *Ophthalmology* 124, 1753-1763. 10.1016/j.ophtha.2017.05.035 28712657 PMC5755466

[BIO048736C17] Dvorak H. F., Brown L. F., Detmar M. and Dvorak A. M. (1995). Vascular permeability factor/vascular endothelial growth factor, microvascular hyperpermeability, and angiogenesis. *Am. J. Pathol.* 146, 1029-1039. 10.1007/978-3-642-59953-8_6 7538264 PMC1869291

[BIO048736C18] Eandi C. M., Ciardella A., Parravano M., Missiroli F., Alovisi C., Veronese C., Morara M. C., Grossi M., Virgili G. and Ricci F. (2017). Indocyanine green angiography and optical coherence tomography angiography of choroidal neovascularization in age-related macular degeneration. *Invest. Ophthalmol. Vis. Sci.* 58, 3690-3696. 10.1167/iovs.17-21941 28738134

[BIO048736C19] Edelman J. L. and Castro M. R. (2000). Quantitative image analysis of laser-induced choroidal neovascularization in rat. *Exp. Eye Res.* 71, 523-533. 10.1006/exer.2000.0907 11040088

[BIO048736C20] Fernandez-Godino R., Bujakowska K. M. and Pierce E. A. (2018). Changes in extracellular matrix cause RPE cells to make basal deposits and activate the alternative complement pathway. *Hum. Mol. Genet.* 27, 147-159. 10.1093/hmg/ddx392 29095988 PMC6251553

[BIO048736C21] Fischer M. D., Huber G., Beck S. C., Tanimoto N., Muehlfriedel R., Fahl E., Grimm C., Wenzel A., Remé C. E., van de Pavert S. A. et al. (2009). Noninvasive, in vivo assessment of mouse retinal structure using optical coherence tomography. *PLoS ONE* 4, e7507 10.1371/journal.pone.0007507 19838301 PMC2759518

[BIO048736C22] Fisher K. J., Jooss K., Alston J., Yang Y., Haecker S. E., High K., Pathak R., Raper S. E. and Wilson J. M. (1997). Recombinant adeno-associated virus for muscle directed gene therapy. *Nat. Med.* 3, 306-312. 10.1038/nm0397-306 9055858

[BIO048736C23] Fuh G., Wu P., Liang W.-C., Ultsch M., Lee C. V., Moffat B. and Wiesmann C. (2006). Structure-function studies of two synthetic anti-vascular endothelial growth factor Fabs and comparison with the Avastin™ Fab. *J. Biol. Chem.* 281, 6625-6631. 10.1074/jbc.M507783200 16373345

[BIO048736C24] Gale N. W., Thurston G., Hackett S. F., Renard R., Wang Q., Mcclain J., Martin C., Witte C., Witte M. H., Jackson D. et al. (2002). Angiopoietin-2 is required for postnatal angiogenesis and lymphatic patterning, and only the latter role is rescued by Angiopoietin-1. *Dev. Cell* 3, 411-423. 10.1016/S1534-5807(02)00217-4 12361603

[BIO048736C25] Gao Y., Yu T., Zhang Y. and Dang G. (2018). Anti-VEGF monotherapy versus photodynamic therapy and anti-VEGF combination treatment for neovascular age-related macular degeneration: a meta-analysis. *Invest. Ophthalmol. Vis. Sci.* 59, 4307-4317. 10.1167/iovs.17-23747 30372759

[BIO048736C26] Gemenetzi M., Lotery A. J. and Patel P. J. (2017). Risk of geographic atrophy in age-related macular degeneration patients treated with intravitreal anti-VEGF agents. *Eye (Lond)* 31, 1-9. 10.1038/eye.2016.208 27716750 PMC5233933

[BIO048736C27] Giani A., Thanos A., Roh M. I., Connolly E., Trichonas G., Kim I., Gragoudas E., Vavvas D. and Miller J. W. (2011). In vivo evaluation of laser-induced choroidal neovascularization using spectral-domain optical coherence tomography. *Invest. Ophthalmol. Vis. Sci.* 52, 3880-3887. 10.1167/iovs.10-6266 21296820

[BIO048736C28] Grossniklaus H. E., Ling J. X., Wallace T. M., Dithmar S., Lawson D. H., Cohen C., Elner V. M., Elner S. G. and Sternberg P. Jr (2002). Macrophage and retinal pigment epithelium expression of angiogenic cytokines in choroidal neovascularization. *Mol. Vis.* 8, 119-126. 11979237

[BIO048736C29] Grossniklaus H. E., Kang S. J. and Berglin L. (2010). Animal models of choroidal and retinal neovascularization. *Prog. Retin. Eye Res.* 29, 500-519. 10.1016/j.preteyeres.2010.05.003 20488255 PMC2962694

[BIO048736C30] Hackett S. F., Wiegand S., Yancopoulos G. and Campochiaro P. A. (2002). Angiopoietin-2 plays an important role in retinal angiogenesis. *J. Cell. Physiol.* 192, 182-187. 10.1002/jcp.10128 12115724

[BIO048736C31] Hanhart J., Comaneshter D. S., Freier-Dror Y. and Vinker S. (2018). Mortality associated with bevacizumab intravitreal injections in age-related macular degeneration patients after acute myocardial infarct: a retrospective population-based survival analysis. *Graefes Arch. Clin. Exp. Ophthalmol.* 256, 651-663. 10.1007/s00417-018-3917-9 29429131

[BIO048736C32] Hasegawa E., Sweigard H., Husain D., Olivares A. M., Chang B., Smith K. E., Birsner A. E., D'amato R. J., Michaud N. A., Han Y. et al. (2014). Characterization of a spontaneous retinal neovascular mouse model. *PLoS ONE* 9, e106507 10.1371/journal.pone.0106507 25188381 PMC4154693

[BIO048736C33] Heiferman M. J. and Fawzi A. A. (2019). Progression of subclinical choroidal neovascularization in age-related macular degeneration. *PLoS ONE* 14, e0217805 10.1371/journal.pone.0217805 31163067 PMC6548359

[BIO048736C34] Hoerster R., Muether P. S., Vierkotten S., Schröder S., Kirchhof B. and Fauser S. (2012). *In-vivo* and *ex-vivo* characterization of laser-induced choroidal neovascularization variability in mice. *Graefes Arch. Clin. Exp. Ophthalmol.* 250, 1579-1586. 10.1007/s00417-012-1990-z 22419036

[BIO048736C35] Hofman P., Blaauwgeers H. G. T., Tolentino M. J., Adamis A. P., Nunes Cardozo B. J., Vrensen G. F. J. M. and Schlingemann R. O. (2000). VEGF-A induced hyperpermeability of blood-retinal barrier endothelium *in vivo* is predominantly associated with pinocytotic vesicular transport and not with formation of fenestrations. *Curr. Eye Res.* 21, 637-645. 10.1076/0271-3683(200008)2121-VFT637 11148600

[BIO048736C36] Huber G., Beck S. C., Grimm C., Sahaboglu-Tekgoz A., Paquet-Durand F., Wenzel A., Humphries P., Redmond T. M., Seeliger M. W. and Fischer M. D. (2009). Spectral domain optical coherence tomography in mouse models of retinal degeneration. *Invest. Ophthalmol. Vis. Sci.* 50, 5888-5895. 10.1167/iovs.09-3724 19661229 PMC2800101

[BIO048736C37] Husain D., Kim I., Gauthier D., Lane A. M., Tsilimbaris M. K., Ezra E., Connolly E. J., Michaud N., Gragoudas E. S., O'neill C. A. et al. (2005). Safety and efficacy of intravitreal injection of ranibizumab in combination with verteporfin PDT on experimental choroidal neovascularization in the monkey. *Arch. Ophthalmol.* 123, 509-516. 10.1001/archopht.123.4.509 15824225

[BIO048736C38] Jo Y.-J., Sonoda K.-H., Oshima Y., Takeda A., Kohno R., Yamada J., Hamuro J., Yang Y., Notomi S., Hisatomi T. et al. (2011). Establishment of a new animal model of focal subretinal fibrosis that resembles disciform lesion in advanced age-related macular degeneration. *Invest. Ophthalmol. Vis. Sci.* 52, 6089-6095. 10.1167/iovs.10-5189 21051730

[BIO048736C39] Johansson A. C., Visse E., Widegren B., Sjögren H.-O. and Siesjö P. (2001). Computerized image analysis as a tool to quantify infiltrating leukocytes: a comparison between high- and low-magnification images. *J. Histochem. Cytochem.* 49, 1073-1079. 10.1177/002215540104900902 11511677

[BIO048736C40] Julien S., Kreppel F., Beck S., Heiduschka P., Brito V., Schnichels S., Kochanek S. and Schraermeyer U. (2008). A reproducible and quantifiable model of choroidal neovascularization induced by VEGF A165 after subretinal adenoviral gene transfer in the rabbit. *Mol. Vis.* 14, 1358-1372. 18682809 PMC2493026

[BIO048736C41] Julien S., Biesemeier A., Taubitz T. and Schraermeyer U. (2014). Different effects of intravitreally injected ranibizumab and aflibercept on retinal and choroidal tissues of monkey eyes. *Br. J. Ophthalmol.* 98, 813-825. 10.1136/bjophthalmol-2013-304019 24457369

[BIO048736C42] Julien-Schraermeyer S., Tschulakow A., Thakkar H., Liu S., Barbara I. and Schraermeyer U. (2019). Stabilization and supporting blood vessel growth as a new concept to treat wet AMD. *Invest. Ophthalmol. Vis. Sci.* 60, 366.

[BIO048736C43] Kaynak S., Kaya M. and Kaya D. (2018). Is there a relationship between use of anti-vascular endothelial growth factor agents and atrophic changes in age-related macular degeneration patients? *Turk. J. Ophthalmol.* 48, 81-84. 10.4274/tjo.27448 29755821 PMC5938481

[BIO048736C44] Kent D. and Sheridan C. (2003). Choroidal neovascularization: a wound healing perspective. *Mol. Vis.* 9, 747-755. 14735062

[BIO048736C45] Kiilgaard J. F., Andersen M. V. N., Wiencke A. K., Scherfig E., LA Cour M., Tezel T. H. and Prause J. U. (2005). A new animal model of choroidal neovascularization. *Acta Ophthalmol. Scand.* 83, 697-704. 10.1111/j.1600-0420.2005.00566.x 16396647

[BIO048736C46] Kokki E., Karttunen T., Olsson V., Kinnunen K. and Ylä-Herttuala S. (2018). Human vascular endothelial growth factor A165 expression induces the mouse model of neovascular age-related macular degeneration. *Genes (Basel)* 9, 438 10.3390/genes9090438 PMC6162490 30200369

[BIO048736C47] Krzystolik M. G., Afshari M. A., Adamis A. P., Gaudreault J., Gragoudas E. S., Michaud N. A., Li W., Connolly E., O'neill C. A. and Miller J. W. (2002). Prevention of experimental choroidal neovascularization with intravitreal anti-vascular endothelial growth factor antibody fragment. *Arch. Ophthalmol.* 120, 338-346. 10.1001/archopht.120.3.338 11879138

[BIO048736C48] Kuroki A. M., Bhutto I. A., Kitaoka T. and Amemiya T. (2002). Natural course of experimental choroidal neovascularization: three-dimensional study with corrosion cast and scanning electron microscope. *Ophthalmic Res.* 34, 200-205. 10.1159/000063886 12297692

[BIO048736C49] Lambert V., Lecomte J., Hansen S., Blacher S., Gonzalez M.-L. A., Struman I., Sounni N. E., Rozet E., DE Tullio P., Foidart J. M. et al. (2013). Laser-induced choroidal neovascularization model to study age-related macular degeneration in mice. *Nat. Protoc.* 8, 2197-2211. 10.1038/nprot.2013.135 24136346

[BIO048736C50] Lebherz C., Maguire A. M., Auricchio A., Tang W., Aleman T. S., Wei Z., Grant R., Cideciyan A. V., Jacobson S. G., Wilson J. M. et al. (2005). Nonhuman primate models for diabetic ocular neovascularization using AAV2-mediated overexpression of vascular endothelial growth factor. *Diabetes* 54, 1141-1149. 10.2337/diabetes.54.4.1141 15793254

[BIO048736C51] Lichtlen P., Lam T. T., Nork T. M., Streit T. and Urech D. M. (2010). Relative contribution of VEGF and TNF-α in the cynomolgus laser-induced CNV model: comparing the efficacy of bevacizumab, adalimumab, and ESBA105. *Invest. Ophthalmol. Vis. Sci.* 51, 4738-4745. 10.1167/iovs.09-4890 20393113

[BIO048736C52] Liu C.-H., Wang Z., Sun Y. and Chen J. (2017). Animal models of ocular angiogenesis: from development to pathologies. *FASEB J.* 31, 4665-4681. 10.1096/fj.201700336R 28739642 PMC5636695

[BIO048736C53] Lu F. and Adelman R. A. (2009). Are intravitreal bevacizumab and ranibizumab effective in a rat model of choroidal neovascularization? *Graefes Arch. Clin. Exp. Ophthalmol.* 247, 171-177. 10.1007/s00417-008-0936-y 18781316

[BIO048736C54] Lyzogubov V. V., Tytarenko R. G., Liu J., Bora N. S. and Bora P. S. (2011). Polyethylene glycol (PEG)-induced mouse model of choroidal neovascularization. *J. Biol. Chem.* 286, 16229-16237. 10.1074/jbc.M110.204701 21454496 PMC3091230

[BIO048736C55] Mori K., Gehlbach P., Ando A., Dyer G., Lipinsky E., Chaudhry A. G., Hackett S. F. and Campochiaro P. A. (2002). Retina-specific expression of PDGF-B versus PDGF-A: vascular versus nonvascular proliferative retinopathy. *Invest. Ophthalmol. Vis. Sci.* 43, 2001-2006. 12037011

[BIO048736C56] Mu H., Wang Y., Chu Y., Jiang Y., Hua H., Chu L., Wang K., Wang A., Liu W., Li Y. et al. (2018). Multivesicular liposomes for sustained release of bevacizumab in treating laser-induced choroidal neovascularization. *Drug Deliv.* 25, 1372-1383. 10.1080/10717544.2018.1474967 29869520 PMC6058521

[BIO048736C57] Nagai N., Lundh von Leithner P., Izumi-Nagai K., Hosking B., Chang B., Hurd R., Adamson P., Adamis A. P., Foxton R. H., Ng Y. S. et al. (2014). Spontaneous CNV in a novel mutant mouse is associated with early VEGF-A-driven angiogenesis and late-stage focal edema, neural cell loss, and dysfunction. *Invest. Ophthalmol. Vis. Sci.* 55, 3709-3719. 10.1167/iovs.14-13989 24845632 PMC4059080

[BIO048736C58] Neary R., Watson C. J. and Baugh J. A. (2015). Epigenetics and the overhealing wound: the role of DNA methylation in fibrosis. *Fibrogenesis Tissue Repair* 8, 18 10.1186/s13069-015-0035-8 26435749 PMC4591063

[BIO048736C59] Neve A., Cantatore F. P., Maruotti N., Corrado A. and Ribatti D. (2014). Extracellular matrix modulates angiogenesis in physiological and pathological conditions. *Biomed. Res. Int.* 2014, 756078 10.1155/2014/756078 24949467 PMC4052469

[BIO048736C60] Oh H., Takagi H., Takagi C., Suzuma K., Otani A., Ishida K., Matsumura M., Ogura Y. and Honda Y. (1999). The potential angiogenic role of macrophages in the formation of choroidal neovascular membranes. *Invest. Ophthalmol. Vis. Sci.* 40, 1891-1898. 10440240

[BIO048736C61] Ohno-Matsui K., Hirose A., Yamamoto S., Saikia J., Okamoto N., Gehlbach P., Duh E. J., Hackett S., Chang M., Bok D. et al. (2002). Inducible expression of vascular endothelial growth factor in adult mice causes severe proliferative retinopathy and retinal detachment. *Am. J. Pathol.* 160, 711-719. 10.1016/S0002-9440(10)64891-2 11839592 PMC1850637

[BIO048736C62] Oshima Y., Oshima S., Nambu H., Kachi S., Hackett S. F., Melia M., Kaleko M., Connelly S., Esumi N., Zack D. J. et al. (2004). Increased expression of VEGF in retinal pigmented epithelial cells is not sufficient to cause choroidal neovascularization. *J. Cell. Physiol.* 201, 393-400. 10.1002/jcp.20110 15389527

[BIO048736C63] Pachydaki S. I., Jakobiec F. A., Bhat P., Sobrin L., Michaud N. A., Seshan S. V. and D'Amico D. J. (2012). Surgical management and ultrastructural study of choroidal neovascularization in punctate inner choroidopathy after bevacizumab. *J. Ophthalmic Inflamm. Infect.* 2, 29-37. 10.1007/s12348-011-0050-x 22120962 PMC3302998

[BIO048736C64] Pennesi M. E., Neuringer M. and Courtney R. J. (2012). Animal models of age related macular degeneration. *Mol. Aspects Med.* 33, 487-509. 10.1016/j.mam.2012.06.003 22705444 PMC3770531

[BIO048736C65] Querques G., Srour M., Massamba N., Georges A., BEN Moussa N., Rafaeli O. and Souied E. H. (2013). Functional characterization and multimodal imaging of treatment-naïve “quiescent” choroidal neovascularization. *Invest. Ophthalmol. Vis. Sci.* 54, 6886-6892. 10.1167/iovs.13-11665 24084095

[BIO048736C66] Roisman L., Zhang Q., Wang R. K., Gregori G., Zhang A., Chen C.-L., Durbin M. K., An L., Stetson P. F., Robbins G. et al. (2016). Optical coherence tomography angiography of asymptomatic neovascularization in intermediate age-related macular degeneration. *Ophthalmology* 123, 1309-1319. 10.1016/j.ophtha.2016.01.044 26876696 PMC5120960

[BIO048736C67] Rolling F., Le Meur G., Stieger K., Smith A. J., Weber M., Deschamps J. Y., Nivard D., Mendes-Madeira A., Provost N., Pereon Y. et al. (2006). Gene therapeutic prospects in early onset of severe retinal dystrophy: restoration of vision in RPE65 Briard dogs using an AAV serotype 4 vector that specifically targets the retinal pigmented epithelium. *Bull. Mem. Acad. R Med. Belg.* 161, 497-508; discussion 508-9. 17503728

[BIO048736C68] Ryan S. J. (1982). Subretinal neovascularization. Natural history of an experimental model. *Arch. Ophthalmol.* 100, 1804-1809. 10.1001/archopht.1982.01030040784015 6182868

[BIO048736C69] Schlingemann R. O. (2004). Role of growth factors and the wound healing response in age-related macular degeneration. *Graefes Arch. Clin. Exp. Ophthalmol.* 242, 91-101. 10.1007/s00417-003-0828-0 14685874

[BIO048736C70] Schmack I., Berglin L., Nie X., Wen J., Kang S. J., Marcus A. I., Yang H., Lynn M. J., Kapp J. A. and Grossniklaus H. E. (2009). Modulation of choroidal neovascularization by subretinal injection of retinal pigment epithelium and polystyrene microbeads. *Mol. Vis.* 15, 146-161. 19158960 PMC2628316

[BIO048736C71] Schmid M. K., Bachmann L. M., Fäs L., Kessels A. G., Job O. M. and Thiel M. A. (2015). Efficacy and adverse events of aflibercept, ranibizumab and bevacizumab in age-related macular degeneration: a trade-off analysis. *Br. J. Ophthalmol.* 99, 141-146. 10.1136/bjophthalmol-2014-305149 25271911

[BIO048736C72] Schraermeyer U. and Julien S. (2013). Effects of bevacizumab in retina and choroid after intravitreal injection into monkey eyes. *Expert Opin Biol. Ther.* 13, 157-167. 10.1517/14712598.2012.748741 23190450

[BIO048736C73] Schraermeyer U., Julien S., Biesemeier A., Bartz-Schmidt K. U. and Wolburg H. (2015). A new kind of labyrinth-like capillary is responsible for leakage from human choroidal neovascular endothelium, as investigated by high-resolution electron microscopy. *Graefes Arch. Clin. Exp. Ophthalmol.* 253, 681-689. 10.1007/s00417-014-2733-0 25042819

[BIO048736C74] Schwesinger C., Yee C., Rohan R. M., Joussen A. M., Fernandez A., Meyer T. N., Poulaki V., Ma J. J. K., Redmond T. M., Liu S. et al. (2001). Intrachoroidal neovascularization in transgenic mice overexpressing vascular endothelial growth factor in the retinal pigment epithelium. *Am. J. Pathol.* 158, 1161-1172. 10.1016/S0002-9440(10)64063-1 11238064 PMC1850362

[BIO048736C75] Semkova I., Peters S., Welsandt G., Janicki H., Jordan J. and Schraermeyer U. (2003). Investigation of laser-induced choroidal neovascularization in the rat. *Invest. Ophthalmol. Vis. Sci.* 44, 5349-5354. 10.1167/iovs.02-0732 14638737

[BIO048736C76] Serra R., Coscas F., Boulet J. F., Cabral D., Lupidi M., Coscas G. J. and Souied E. H. (2019). Predictive activation biomarkers of treatment-naive asymptomatic choroidal neovascularization in age-related macular degeneration. [published online ahead of print, 2019 June 21] *Retina*. 10.1097/IAE.0000000000002604 31259809

[BIO048736C77] Spilsbury K., Garrett K. L., Shen W.-Y., Constable I. J. and Rakoczy P. E. (2000). Overexpression of vascular endothelial growth factor (VEGF) in the retinal pigment epithelium leads to the development of choroidal neovascularization. *Am. J. Pathol.* 157, 135-144. 10.1016/S0002-9440(10)64525-7 10880384 PMC1850220

[BIO048736C78] Stern J. and Temple S. (2015). Retinal pigment epithelial cell proliferation. [published online ahead of print, 2019 June 21] *Exp. Biol. Med. (Maywood)* 240, 1079-1086. 10.1177/1535370215587530 26041390 PMC4935281

[BIO048736C79] Tahiri H., Omri S., Yang C., Duhamel F., Samarani S., Ahmad A., Vezina M., Bussières M., Vaucher E., Sapieha P. et al. (2016). Lymphocytic microparticles modulate angiogenic properties of macrophages in laser-induced choroidal neovascularization. *Sci. Rep.* 6, 37391 10.1038/srep37391 27874077 PMC5118818

[BIO048736C80] Treister A. D., Nesper P. L., Fayed A. E., Gill M. K., Mirza R. G. and Fawzi A. A. (2018). Prevalence of subclinical CNV and choriocapillaris nonperfusion in fellow eyes of unilateral exudative AMD on OCT angiography. *Transl. Vis. Sci. Technol.* 7, 19 10.1167/tvst.7.5.19 PMC6166896 30280004

[BIO048736C81] Wang F., Rendahl K. G., Manning W. C., Quiroz D., Coyne M. and Miller S. S. (2003). AAV-mediated expression of vascular endothelial growth factor induces choroidal neovascularization in rat. *Invest. Ophthalmol. Vis. Sci.* 44, 781-790. 10.1167/iovs.02-0281 12556414

[BIO048736C82] Weisel J. W. and Litvinov R. I. (2017). Fibrin Formation, Structure and Properties. *Subcell. Biochem.* 82, 405-456. 10.1007/978-3-319-49674-0_13 28101869 PMC5536120

[BIO048736C83] Xiong W., Wu D. M., Xue Y., Wang S. K., Chung M. J., Ji X., Rana P., Zhao S. R., Mai S. and Cepko C. L. (2019). AAV cis-regulatory sequences are correlated with ocular toxicity. *Proc. Natl. Acad. Sci. USA* 116, 5785-5794. 10.1073/pnas.1821000116 30833387 PMC6431174

[BIO048736C84] Yi X., Ogata N., Komada M., Yamamoto C., Takahashi K., Omori K. and Uyama M. (1997). Vascular endothelial growth factor expression in choroidal neovascularization in rats. *Graefes Arch. Clin. Exp. Ophthalmol.* 235, 313-319. 10.1007/BF01739641 9176680

[BIO048736C85] Yu L., Wu X., Cheng Z., Lee C. V., Lecouter J., Campa C., Fuh G., Lowman H. and Ferrara N. (2008). Interaction between bevacizumab and murine VEGF-A: a reassessment. *Invest. Ophthalmol. Vis. Sci.* 49, 522-527. 10.1167/iovs.07-1175 18234994

[BIO048736C86] Zhang S., Wu J., Wu X., Xu P., Tian Y., Yi M., Liu X., Dong X., Wolf F., Li C. et al. (2012). Enhancement of rAAV2-mediated transgene expression in retina cells in vitro and in vivo by coadministration of low-dose chemotherapeutic drugs. *Invest. Ophthalmol. Vis. Sci.* 53, 2675-2684. 10.1167/iovs.11-8856 22427581

