# Supplementary material for: A new rat model of treatment-naive quiescent choroidal neovascularization induced by human VEGF165 overexpression
Source: Biol Open. 2020 Jun 11;9(6):bio048736. doi: 10.1242/bio.048736 (PMC7295592; doi:10.1242/bio.048736)
Supplement: Supplementary information [file biolopen-9-048736-s1.pdf]

Supplementary figures and tables

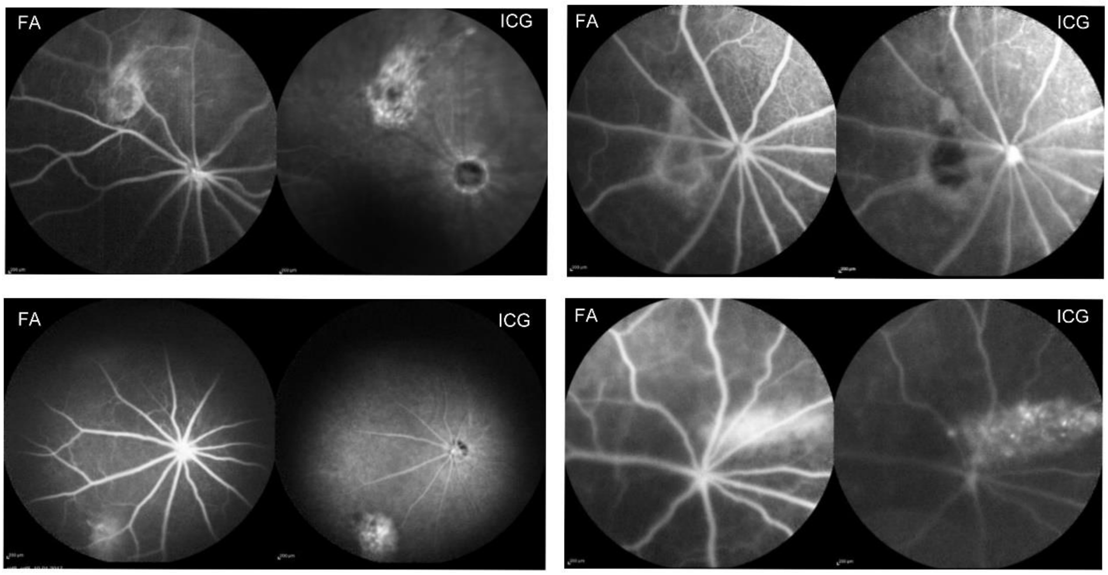

Figure S1. Angiography of four CNV eyes six weeks after AAV-VEGF transduction. ICG shows a larger CNV area than FA in each eye. AAV: adeno-associated virus, CNV: choroidal neovascularization, FA: fluorescein angiography, ICG: indocyanine green angiography.

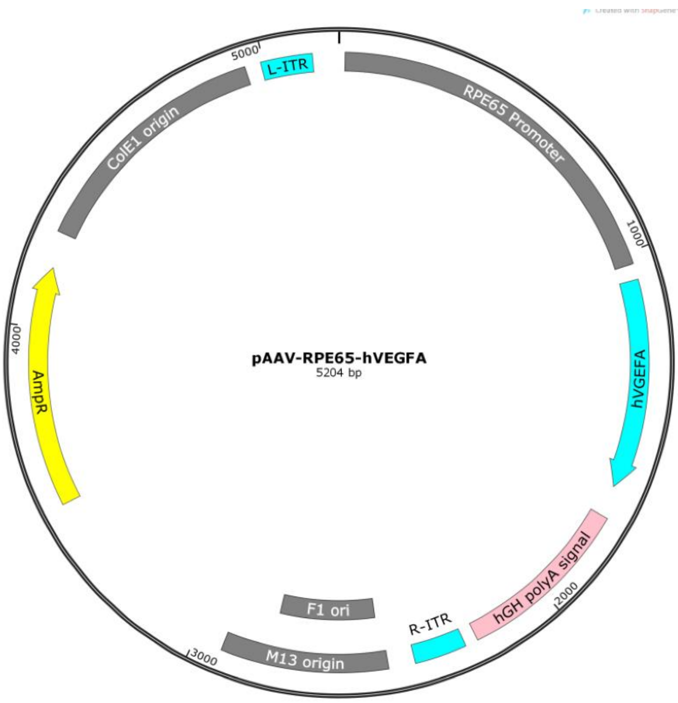

Figure S2. Vector map of the AAV-hVEGF-A165 vector. The AAV vector contains an RPE specific RPE65 promotor instead of the unspecific CMV promotor used in Ad. Vector. AAV: adeno-associated virus.

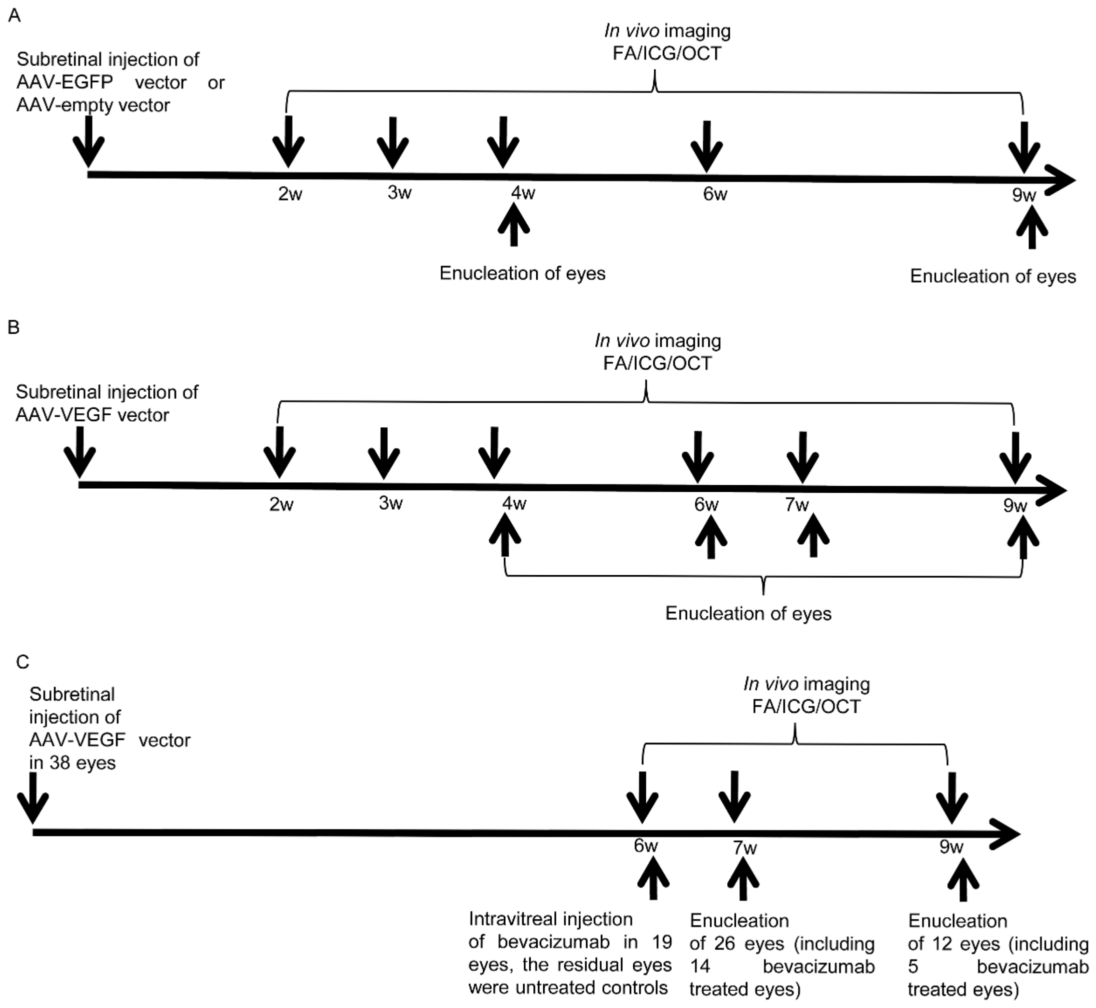

Figure S3. Experimental designs of this study. (A) Vector controls, (B) VEGF overexpression, (C) Treatment study. The eyes were enucleated immediately after *in vivo* imaging examinations. AAV: adeno-associated virus, EGFP: enhanced green fluorescent protein, FA: fluorescein angiography, ICG: indocyanine green angiography, OCT: optical coherence tomography, w: weeks.

Biology Open • Supplementary information

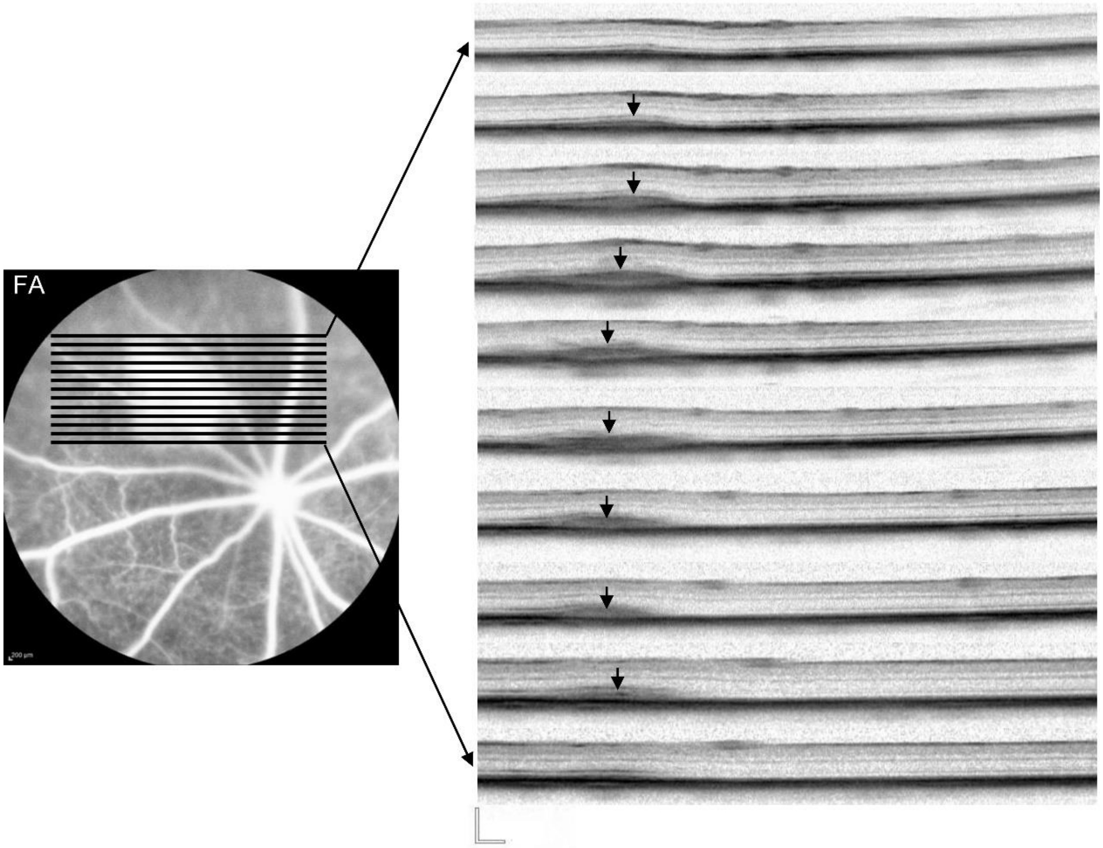

Figure S4. Volume scan of one eye. The lesion in each OCT image (right images) is marked with an arrow and corresponds to the hyperfluorescent region in the FA (left image). OCT: optical coherence tomography. Scale bar: 200 μm.

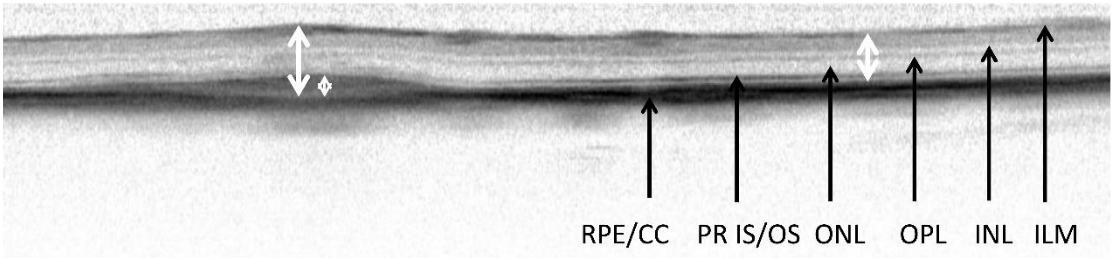

Figure S5. Measurement of the thickness of the retina and CNV lesion. The different layers of the retina were marked with black arrows, respectively. The middle white double-headed arrow marks the thickness of the CNV lesion, the left one is the thickness of the retina and contains the CNV lesion, and the right one is the normal retinal thickness. CC: choriocapillaris, CNV: choroidal neovascularization, INL: inner nuclear layer, ILM: internal limiting membrane, ONL: outer nuclear layer, OPL: outer plexiform layer, PR IS: photoreceptor inner segments, PR OS: photoreceptor outer segments, RPE: retinal pigment epithelium.

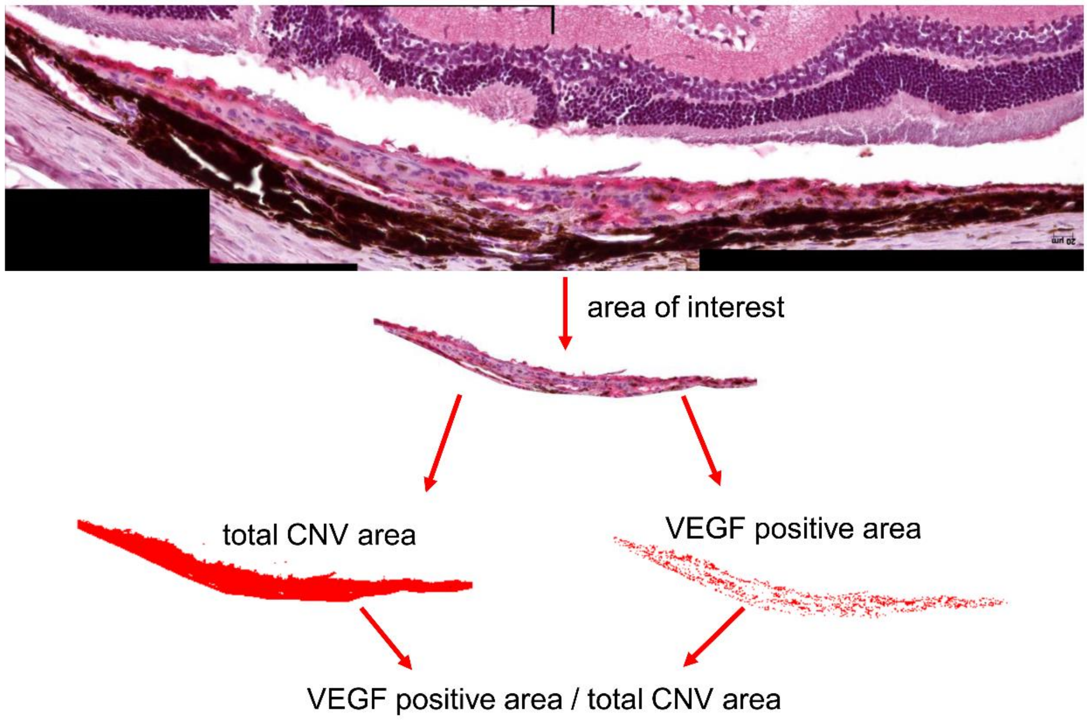

Figure S6. Quantification of VEGF expression in CNV eyes. First, the area of interest is obtained from the original image. Then the total CNV area and the VEGF positive area can be measured by using ImagePro Plus 6.0. Finally, the percentage of VEGF positive area of the total CNV area is calculated.

Table S1. Number of eyes with CNV detected by *in vivo* imaging.

<table>
<tr><td rowspan="4">Injection reagent</td><td colspan="4">Number of CNV eyes/Number of total eyes (Percentage of CNV eyes)</td></tr>
<tr><td colspan="2" rowspan="2">Duration of VEGF vector transduction (eyes without bevacizumab treatment)</td><td colspan="2">bevacizumab treated eyes</td></tr>
<tr><td colspan="2">Duration of treatment</td></tr>
<tr><td>4w</td><td>6w-9w</td><td>1w</td><td>3w</td></tr>
<tr><td>AAV-VEGF vector</td><td>3/6 (50%)</td><td>29/32 (91%)</td><td>14/14 (100%)</td><td>5/5 (100%)</td></tr>
<tr><td>Total</td><td colspan="4">4w: 50%; 6w: 93% (including eyes treated with bevacizumab)</td></tr>
</table>

Biology Open • Supplementary information

Table S2. Number of eyes injected with AAV-VEGF vectors, control AAV vectors (EGFP, empty). AAV: adeno-associated virus, EGFP: enhanced green fluorescent protein.

Injection reagent	Number of eyes					
	Duration of vector transduction (eyes without bevacizumab treatment)				Bevacizumab treated eyes<br>Duration of treatment	
	4w	6w	7w	9w	1w	3w
AAV-VEGF vector	6	4	12	16	14	5
AAV-EGFP vector	5			1		
AAV-empty vector	3					
Total	66					

Biology Open • Supplementary information
